# The genomic landscape of lung cancer in never-smokers from the Women’s Health Initiative

**DOI:** 10.1172/jci.insight.174643

**Published:** 2024-07-25

**Authors:** Sitapriya Moorthi, Amy Paguirigan, Pushpa Itagi, Minjeong Ko, Mary Pettinger, Anna C.H. Hoge, Anwesha Nag, Neil A. Patel, Feinan Wu, Cassie Sather, Kevin M. Levine, Matthew P. Fitzgibbon, Aaron R. Thorner, Garnet L. Anderson, Gavin Ha, Alice H. Berger

**Affiliations:** 1 Human Biology Division,; 2 Clinical Research Division, and; 3 Public Health Sciences Division, Fred Hutchinson Cancer Center, Seattle, Washington, USA.; 4 Center for Cancer Genomics, Dana-Farber Cancer Institute, Boston, Massachusetts, USA.; 5 Genomics and Bioinformatics Shared Resource, Fred Hutchinson Cancer Center, Seattle, Washington, USA.; 6 Division of Hematology and Oncology, Department of Medicine and; 7 Department of Genome Sciences, University of Washington, Seattle, Washington, USA.

**Keywords:** Genetics, Therapeutics, Genetic variation, Lung cancer, Molecular diagnosis

## Abstract

Over 200,000 individuals are diagnosed with lung cancer in the United States every year, with a growing proportion of cases, especially lung adenocarcinoma, occurring in individuals who have never smoked. Women over the age of 50 comprise the largest affected demographic. To understand the genomic drivers of lung adenocarcinoma and therapeutic response in this population, we performed whole genome and/or whole exome sequencing on 73 matched lung tumor/normal pairs from postmenopausal women who participated in the Women’s Health Initiative. Somatic copy number alterations showed little variation by smoking status, suggesting that aneuploidy may be a general characteristic of lung cancer regardless of smoke exposure. Similarly, clock-like and APOBEC mutation signatures were prevalent but did not differ in tumors from smokers and never-smokers. However, mutations in both *EGFR* and *KRAS* showed unique allelic differences determined by smoking status that are known to alter tumor response to targeted therapy. Mutations in the MYC-network member *MGA* were more prevalent in tumors from smokers. Fusion events in *ALK*, *RET*, and *ROS1* were absent, likely due to age-related differences in fusion prevalence. Our work underscores the profound effect of smoking status, age, and sex on the tumor mutational landscape and identifies areas of unmet medical need.

## Abstract 2

The genomic landscape of lung cancer in older never-smoker women has unique patterns of mutations but is indistinguishable in its copy number alterations from smokers.

## Graphical abstract

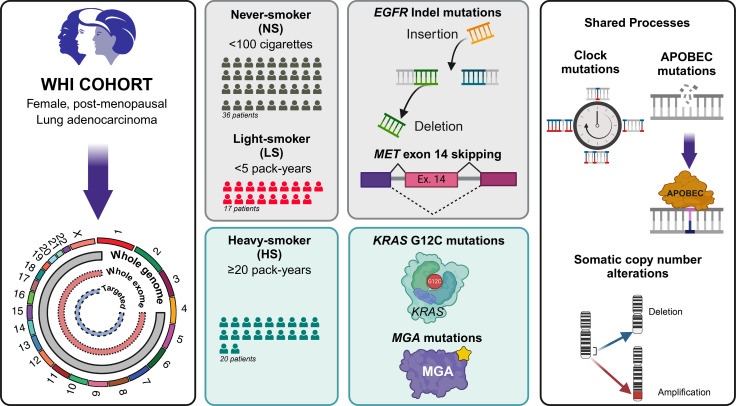

## Introduction

Lung cancer is the deadliest form of cancer in both men and women, with lung adenocarcinoma being the most prevalent subtype (1). The discovery of driver oncogenes such as *EGFR* and the development of oncogene-targeted therapies transformed the clinical management of lung adenocarcinoma over the last decade (2). These discoveries changed the clinical course of lung cancer and contributed to the recent decline in lung cancer mortality (3). However, much of the genetic characterization of lung cancer has been performed on tumors from patients with a history of smoking (4, 5). Cigarette smoking is the primary risk factor for developing lung cancer. However, 10%–15% of lung cancer cases in the United States, and up to 20% of cases worldwide, occur in patients who have never smoked, defined as individuals who have smoked fewer than 100 cigarettes in their lifetime (6–8). In recent years, the percentage of lung cancer cases in never-smokers has increased to 17% in men and 24% in women, which may reflect both a decrease in global smoking behavior and an increase in the incidence of lung cancer in never-smokers (9). A particular concern is that the incidence of lung cancer in women appears higher than in men, even after controlling for differences in smoking behavior (10). If considered as a separate disease, lung cancer in never-smokers would be the seventh largest cause of death due to cancer (6, 11). Thus, even as smoking rates decline, lung cancer in never-smokers is expected to contribute a substantial cancer burden in the United States and worldwide.

Lung cancer in never-smokers is distinct from lung cancer in smokers due to many unique genetic and clinical characteristics (12, 13). The most frequently diagnosed histological subtype of lung cancer in never-smokers is adenocarcinoma (6), that women are diagnosed more often than men (14, 15), and that the majority of lung cancer cases in South and East Asian women occur in never-smokers (6). Although lung cancer in never-smokers is, on average, diagnosed at a younger age than cases in smokers (16), the majority of cases occur in individuals older than 50 (17).

At the genetic level, lung tumors from smokers have a significantly higher overall somatic mutation rate and different somatic mutation patterns than tumors from never-smokers, suggesting alternative mechanisms of cancer development (5, 18). Tumors from never-smokers have a higher prevalence of *EGFR* mutations and fusions involving *ALK*, *RET*, *ROS1*, or *NRG1* (19–21) and have fewer *KRAS* mutations than tumors from smokers (22). The NCI SHERLOCK study shows that tumors from never-smokers cluster into distinct groups based on arm-level copy number alterations, which correlate with prognosis (12). However, because these subtypes were defined primarily from tumors from never-smokers, it remains unclear if copy number subtypes are unique to never-smokers or are a general characteristic of lung cancer.

Here, we sought to define the genetic landscape of lung adenocarcinoma tumors from postmenopausal female never-smokers. We performed whole exome and/or whole genome sequencing of tumor and matched normal DNA from Women’s Health Initiative (WHI) participants who developed lung cancer (23). We find that never-smokers display a unique mutational spectrum of *EGFR* and *KRAS* variants with implications for both targeted and immunotherapy response. Chromosomal fusions in *ALK*, *RET*, and *ROS1* were surprisingly absent, suggesting that lung cancers from older female never-smokers may have lower rates of these fusion oncogenes. Somatic mutation signature analysis found DNA repair defect signatures in 22% of the tumors, although we could not attribute this phenotype to germline cancer predisposition variants. Finally, we confirm the recent finding of distinct copy number subtypes of lung adenocarcinoma (12), but we find that these subtypes are shared across tumors from both smokers and never-smokers, indicating that aneuploidy and somatic copy number alteration (SCNA) are general features of lung cancer not related to smoking.

## Results

### Genomic profiling of lung adenocarcinomas in female never-smokers.

The majority of lung cancer cases in never-smokers occur in women older than 50 (Supplemental Figure 1A; supplemental material available online with this article; https://doi.org/10.1172/jci.insight.174643DS1) (17). Clinical and molecular characteristics of lung cancer appear to differ between younger and older lung cancer patients (16, 24, 25). The WHI provides a unique opportunity to understand the biology and risk factors of lung cancer in postmenopausal women. The WHI was initially conceived as 3 overlapping clinical trials and an observational study to evaluate risk factors for cancer and cardiovascular disease (23, 26). Beginning in the 1990s, the WHI prospectively enrolled over 160,000 women at 40 different centers. Many women in the study went on to develop cancer, including lung cancer. We requested tumor tissue and matched normal blood DNA to profile the genetic landscape of lung cancer in postmenopausal WHI participants (Table 1 and Supplemental Table 1). We chose to enrich the study cohort for women with fewer than 100 lifetime cigarettes (never-smokers) or those with a light smoking history of fewer than 5 pack-years. A smaller group of heavy smokers with greater than 20 pack-year smoking history were matched to this cohort on cancer stage, diagnosis year, and tumor purity. The median age at diagnosis of women in the study was 73, 76.5, and 77 years for heavy smokers, light smokers, and never-smokers (Table 1). The majority of cases were adenocarcinoma not otherwise specified (NOS) (*n* = 36), but several histologic subtypes of adenocarcinoma were also represented including acinar (*n* = 13), lepidic (*n* = 11), and colloidal (*n* = 6), among others (Table 1). Because samples were taken from surgical resections, the tumors were mostly from cases that were localized or had only regional spread (Table 1).

Tumor histology was reviewed by a centralized pathologist (Peggy Porter, Fred Hutchinson Cancer Center), and tumors were enriched for tumor content by macrodissection prior to sequencing. To identify mutated genes and SCNAs, extracted DNA from tumor and matched normal blood was subjected to whole exome sequencing (WES) using a custom “exome-plus” bait set used for clinical WES (Methods). This bait set includes coverage in intronic regions frequently involved in chromosomal rearrangements such as *ALK*, *RET*, and *ROS1* (27). In total, 73 tumor/normal pairs from 56 never-/light smokers and 17 heavy smokers passed quality control assessment and were used for downstream analysis (Supplemental Table 2). Tumors and normal samples were sequenced to a median target coverage of 93× or 80×, respectively.

### Unique prevalence of somatically mutated genes in tumors from smokers and never-smokers.

**Figure 1:**
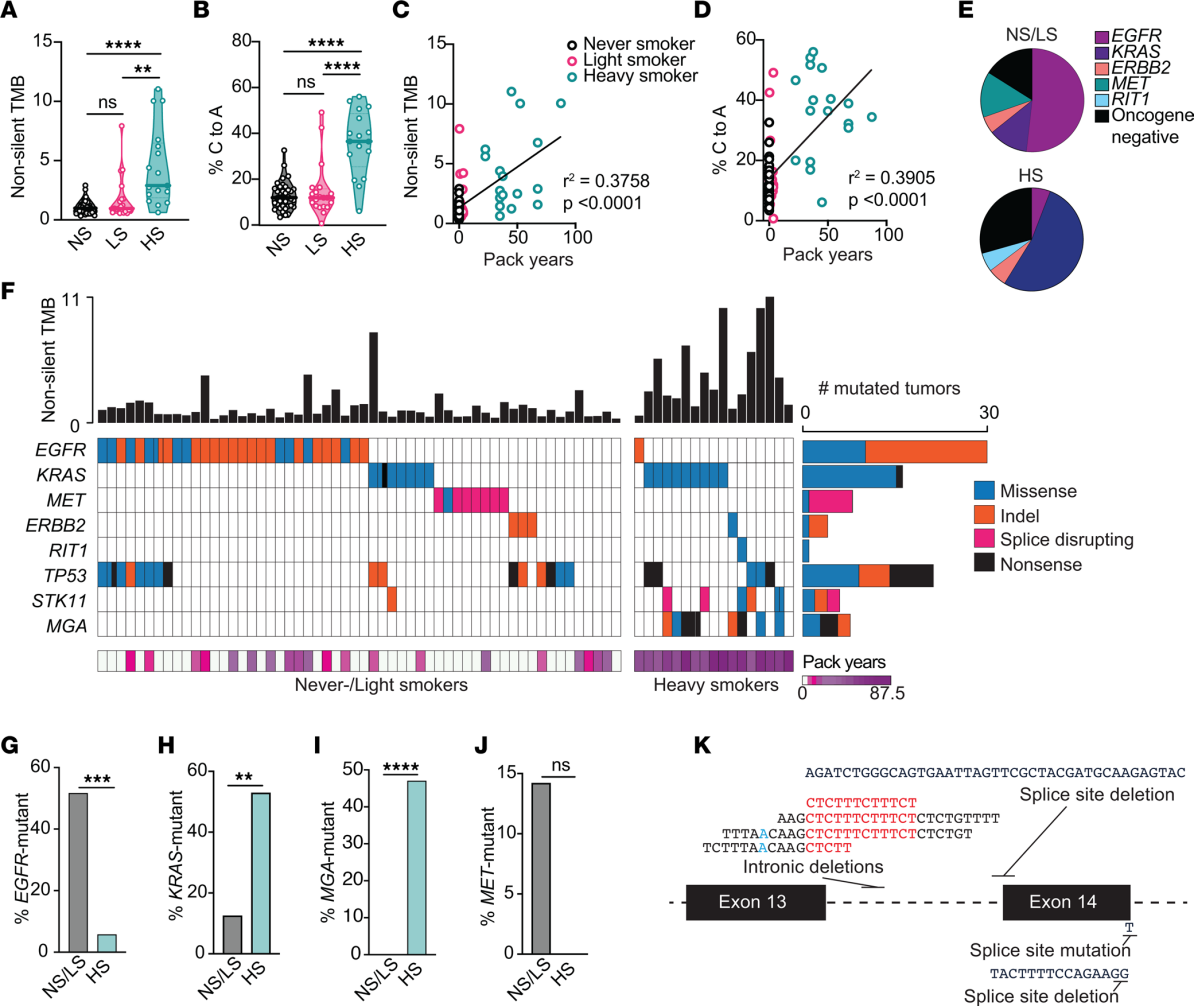
Unique prevalence of somatically mutated genes in tumors from smokers and never-smokers. (**A**) Nonsilent tumor mutational burden (TMB) rate (mutations/Mbp) in never-smokers (NS; < 100-lifetime cigarettes), light smokers (LS; < 5 pack years), and heavy smokers (HS; > 20 pack years). (**B**) Percent of C to A transversions. (**C**) Association between nonsilent TMB and pack-years smoked. (**D**) Association between percent C to A transversions and pack years smoked. (**E**) Prevalence of canonical Ras/RTK pathway driver mutations in never-/light smokers (NS/LS) and heavy smokers (HS). (**F**) Oncoplot of highlighted mutated genes. Each column is an individual tumor. The top bar plot shows the nonsilent TMB rate (mutations/Mbp) for each tumor. (**G**–**J**) The total number of samples with *EGFR* (**G**), *KRAS* (**H**), *MGA* (**I**), and *MET* (**J**) mutations in NS/LS versus HS. (**K**) Schematic representation of the *MET* locus between exon 13 and exon 15 and identified alterations likely to promote exon 14 skipping. Blue nucleotides represent branchpoint site, and red nucleotides represent the polypyrimidine tract. ****P < 0.0001; ***P < 0.001; **P < 0.01; Kruskal-Wallis/Dunn’s test (**A** and **B**), simple linear regression (**C** and **D**), or 2-tailed Fisher’s exact test (**G**–**J**).

The single nucleotide variant (SNV) and insertion/deletion (indel) landscapes of tumors from never- and light smokers showed extensive differences compared with those from heavy smokers. Tumors from heavy smokers had a significantly higher nonsilent tumor mutational burden (TMB) and a greater percentage of C to A transversion mutations compared with tumors from never-smokers (Figure 1, A and B), consistent with cigarette smoke being a direct mutagen of the genome (4, 5, 18). Moreover, smoke exposure, measured by the pack-years of cigarette smoked, significantly correlated with both TMB (*r*^2^ = 0.3758) and percent of C to A transversions (*r*^2^ = 0.3905) (Figure 1, C and D). We noted that tumors from never- and light smokers had indistinguishable TMB and C to A mutation rates (Figure 1, A and B) and, thus, were grouped for subsequent analyses.

We next explored if sex or age had any influence on the TMB. Analysis of external data from TCGA (28), Memorial Sloan-Kettering Cancer Center (MSK) (29–32), and the AACR GENIE project (33–35) showed increased nonsilent TMB in patients with a history of smoking, whereas age and sex did not consistently affect the nonsilent TMB after controlling for smoking status (Supplemental Figure 1, B–G).

Mutations in the receptor tyrosine kinase/Ras/Raf (RTK/Ras/Raf) pathway are critical drivers of lung adenocarcinoma (5). In total, 84% of tumors from never-/light smokers and 71% of heavy smokers (Figure 1E) had mutations in canonical drivers of this pathway. However, the proportion of samples with mutations in specific genes of the pathway varied between the groups (Figure 1F and Supplemental Tables 3 and 4). *EGFR* mutations were more prevalent in never-/light smokers (52% versus 6%; Fisher’s exact test, *P* = 0.0006) (Figure 1G) and *KRAS* mutations were enriched in heavy smokers (13% versus 53%; Fisher’s exact test, *P* = 0.0012; Figure 1H).

Mutations in the MYC transcription factor network tumor suppressor gene *MGA* were previously identified as inactivating mutations in 10% of lung adenocarcinomas (5). MGA regulates MYC-mediated transcription via its ability to dimerize with MAX and recruit a variant Polycomb complex (36). We have previously identified *MGA* as a driver event that cooperates with mutant *KRAS* to promote lung cancer in vivo (37). Unexpectedly, we observed that *MGA* mutation was significantly associated with smoking history and extremely prevalent in smokers in our study (47% of smokers; Figure 1, F and I), cooccurring with mutant *KRAS* in 4/8 *MGA*-mutant tumors (Figure 1F).

To our knowledge, the enrichment of *MGA* mutations in tumors from heavy smokers is a novel observation, so to verify this finding, we analyzed existing data from 3 large cohorts. Data from TCGA, MSK, and GENIE (28–35) confirmed that *MGA* mutations are more prevalent in tumors from individuals with a history of smoking in both men and women (Supplemental Figure 2, A–D) and in both younger and older patients (Supplemental Figure 2, E–H). Together, these data identify tumor suppressor inactivation of *MGA* as a highly recurrent contributor to smoking-associated lung cancer.

In recent years, somatic *MET* exon 14 skipping mutations have emerged as biomarkers for clinical response to *MET*-targeted therapies (5, 38, 39). These variants disrupt the splice sites flanking exon 14, resulting in exon skipping and expression of a smaller isoform of *MET* with enhanced protein stability and kinase activity (40, 41). We observed *MET* exon 14 skipping mutations at a higher prevalence than previous studies (5, 38), with a trend toward exclusivity to the never-/light smoker group (8 of 56 [14%] versus 0 of 17 [0%] in heavy smokers; Fisher’s exact test, *P* = 0.18) (Figure 1J). In addition to SNVs and deletions that disrupt canonical splice site motifs, intronic baits included in our exome panel allowed us to identify deletions in the upstream intron encompassing the intron 13–14 branchpoint or the polypyrimidine tract in 4 tumors (40, 41) (Figure 1K). Analysis of the TCGA, MSK, and GENIE cohorts confirmed that *MET* splicing mutations are significantly enriched in never-/light/moderate smokers compared with heavy smokers/ever-smokers (Supplemental Figure 2, I–L). Moreover, *MET* mutations were more frequent in tumors from patients diagnosed above age 50 compared with tumors from younger patients (Supplemental Figure 2, M–P).

Several additional non-Ras pathway genes showed altered mutational burden in smokers and never-/light smokers (Supplemental Figure 3, A and B). These genes included *STK11*, also known as *LKB1*, which is known to have increased prevalence in smokers (42) and confer a worse prognosis for tumors treated with PD-1/PD-L1 checkpoint inhibitors (43). *STK11* alterations occurred more frequently in heavy smokers than never-/light smokers (29% versus 2%, Fisher’s exact test, *P* = 0.0021). We also noted the enrichment of *ATF7IP* somatic mutations in tumors from smokers (3 of 17 [18%] versus 0 of 56 [0%]; Fisher’s exact test, *P* = 0.01) (Supplemental Figure 3B). However, only 3 overall somatic mutations in *ATF7IP* were identified, and *ATF7IP* mutations have been previously observed in never-smokers (44).

Fusions involving *RET*, *ROS1*, or *ALK* have been reported to frequently occur in lung tumors from never-smokers, so we designed the custom WES panel to include baits that cover breakpoints in *ALK*, *RET*, and *ROS1*. However, analysis using SvABA (45) did not identify any *ALK*, *RET*, or *ROS1* fusions. As an orthogonal validation, we employed an RNA-based amplicon sequencing method to search for fusions in RNA from 5 oncogene-negative samples and 2 positive control samples with known fusions (Methods). A *KIF5B-RET* and *EML4-ALK* fusion were readily detected in the positive control patient-derived xenograft and cell line, but no fusions were detected in 5 of 5 tumor samples (Supplemental Figure 4A). Moreover, whole genome sequencing of 10 of the oncogene negative samples (2 never-smoker samples, 4 light smoker samples, and 4 heavy smoker samples) (Supplemental Figure 4B and Supplemental Tables 5 and 6) identified numerous intra- and interchromosomal structural variants (SVs) (median intrachromosomal events = 8 and interchromosomal = 18). However, no fusions in *ALK*, *RET*, or *ROS1* were identified, confirming the absence of these alterations in this cohort.

We analyzed data from 6 external cohorts (4, 5, 12, 30, 32, 46) including 2,059 patient samples profiled for SVs to understand whether this absence in fusion positive tumors correlated with age, sex, or stage of diagnosis. Analysis of the external cohorts identified 122 patients (0.6%) with oncogenic fusions involving *ALK*, *RET*, or *ROS1*. Samples with fusions were significantly enriched in those with no or low smoking history compared with heavy or ever smokers (Supplemental Figure 5A) regardless of sex (Supplemental Figure 5B). However, fusions were significantly enriched in patients diagnosed at less than 50 years of age compared with those diagnosed at or above age 50 (Supplemental Figure 5C). This finding is consistent with inference from mutational timing analysis, which showed that chromosomal fusion events likely occur early in life and lead to early-onset lung cancer (47). One other explanation as to a lack of these fusions in the WHI cohort could be that most of the samples come from early-stage disease (Supplemental Figure 5D). Analysis of external cohorts shows an increasing trend of fusion-positive samples with increasing disease stage (Supplemental Figure 5E). Thus, the absence of fusions in this study likely reflects the unique patient and tumor characteristics of this cohort, including the advanced age at diagnosis and early stage of the tumors profiled.

### Mutational processes of tumors in never-smokers include clock, APOBEC, and DDR deficiency.

**Figure 2:**
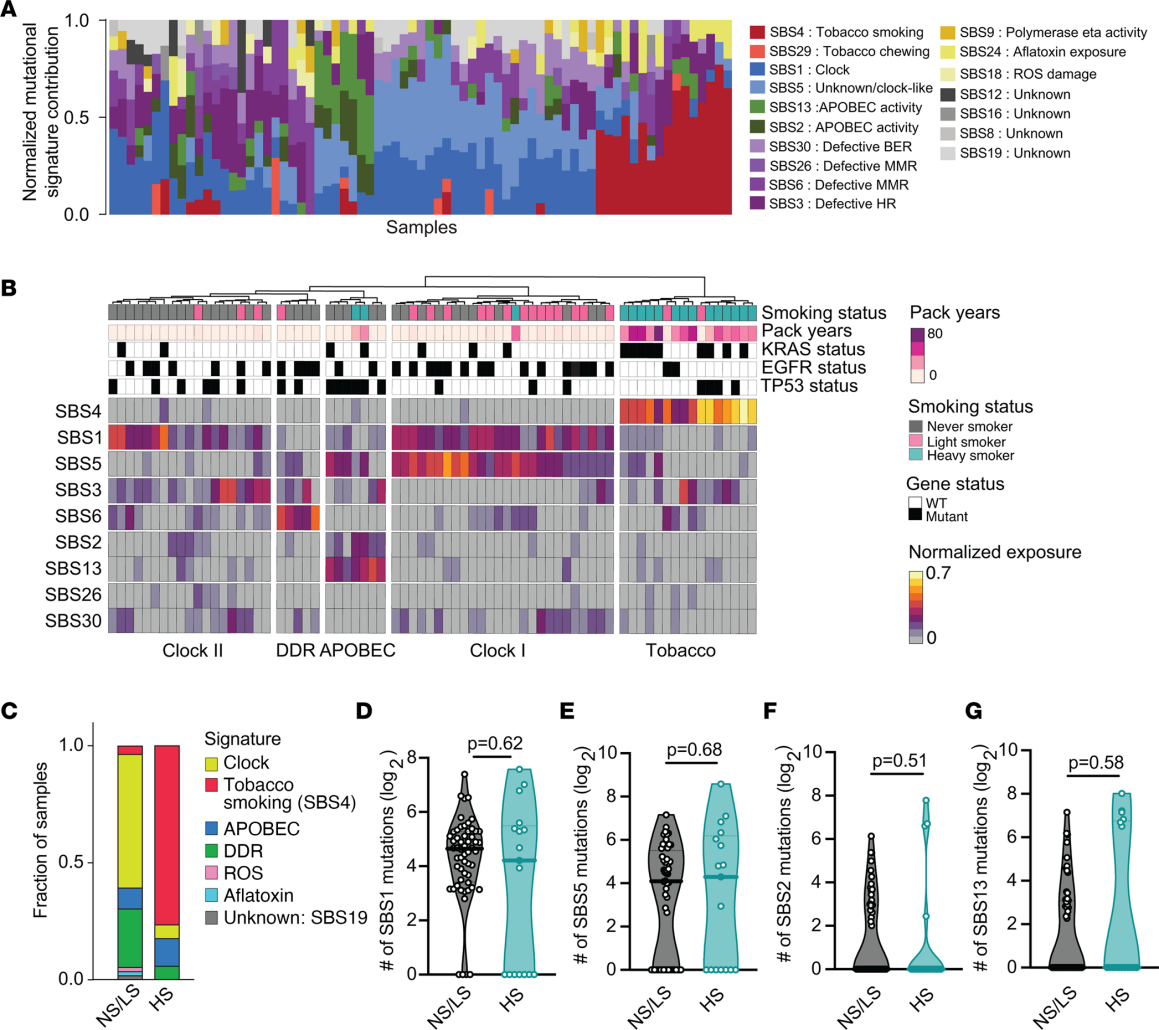
Somatic mutational signatures distinguish tumors from never-/light and heavy smokers. (**A**) Contribution of each SBS mutational signature to the total repertoire of mutations in each tumor. The fractional contribution is calculated by normalizing each signature exposure to the total signature exposure in each tumor. Each stacked bar represents 1 tumor. (**B**) Heatmap of unsupervised clustering of 9 normalized mutational signatures using Ward’s minimum variance method for both samples and signatures. The clustering is based on the normalized signature exposures. (**C**) Stacked bar graph indicating the mutational signature contributing to the maximal mutational burden for each sample. (**D**–**G**) Comparison of the estimated absolute number of mutations attributable to clock signatures (SBS1 and SBS5) and APOBEC signatures (SBS2 and SBS13) in never-/light smokers and heavy smokers. Exact *P* values are shown for testing by Mann-Whitney *U* test (2-tailed).

Mutational signatures in cancer provide insight into cancer etiology and mechanisms of tumor therapy response (48). Single base substitution (SBS) mutational signatures have been defined by classifying mutations according to the base change (e.g., C to T) and the flanking upstream and downstream base context (48). We applied established methods to determine the contribution of known signatures from COSMIC (https://cancer.sanger.ac.uk/signatures/) to each tumor somatic mutational profile. We identified 17 signatures that contributed to mutational profiles, accounting for a median of 90% of the mutations in each sample (Figure 2A and Supplemental Table 7). The predominant signatures were tobacco (SBS4), age-related clock-like process (SBS1 and SBS5), defective DNA damage response (SBS3, SBS6, SBS26, SBS30), and APOBEC mutagenesis (SBS2 and SBS13) (Figure 2A). Several previously described signatures of unknown etiology were identified, but the contribution of these signatures to each mutational profile was low (about 3% of all somatic SNVs).

Unsupervised hierarchical clustering of mutational signature exposure identified 5 predominant mutational signature groups (Figure 2B). As expected, the tobacco/SBS4-high group included 14 of 17 (82%) heavy smokers, with SBS4 as the dominant mutagenic process accounting for an average of 41% of all somatic mutations in tumors of this group. Two tumors from light smokers also clustered into this group; one had an *EGFR* mutation and the other had a *KRAS* mutation. The remaining 54 never- and light smokers had little evidence of smoke exposure (SBS4 minimum and median fraction contribution = 0), despite most individuals in the study reporting passive smoke exposure (Supplemental Figure 6A). These data suggest that passive smoke exposure is not likely to be a driver of mutagenesis in lung cancer in never-smokers.

Clock-like and DNA damage signatures dominated the mutagenic landscape of tumors in never-/light smokers (Figure 2, A and B), accounting for a higher proportion of mutations in each sample than in heavy smokers. Clock-like signatures SBS1 and SBS5 were the predominant mutagenic process in 33 of 56 (59%) never-/light smokers (Figure 2C). These clock-like signatures are believed to arise from mitotic errors, with the accumulation of clock-like mutations increasing with age (49). We observed a significant correlation between SBS1 and SBS5 signatures (simple linear regression *r*^2^ = 0.1208; *P* = 0.0026), but a group of SBS5-low tumors was also evident, indicating that the 2 signatures may reflect related but distinct mutagenic processes (Supplemental Figure 6B).

Seven tumors with elevated SBS2 and SBS13 signatures clustered in an APOBEC-high cluster (Figure 2, B and C). This group comprised 5 never-/light and 2 heavy smokers and was enriched for samples with mutant *TP53* (6 of 7; Fisher’s exact test, *P* = 0.0018). The APOBEC mutational signature is characterized by C to T and C to G mutations believed to be induced by elevated activity of APOBEC enzymes with a polynucleotide cytosine deaminase activity (50–52). APOBEC activity has been shown to be associated with the early onset of lung adenocarcinoma in female never-smokers of East Asian ancestry (53).

To determine whether never-smokers had a higher overall burden of clock and APOBEC mutations, we estimated the absolute number of mutations attributable to each signature. This analysis revealed no significant difference in clock-like mutagenesis (SBS1, *P* = 0.62; SBS5, *P* = 0.68; Mann-Whitney *U* test) or APOBEC mutagenesis in tumors from never-/light and heavy smokers (*P* = 0.51 and *P* = 0.58 for SBS1 and SBS5, respectively; Mann-Whitney *U* test) (Figure 2, D–G). Therefore, we conclude that these common mutagenic processes are operative in lung cells in general rather than disproportionately affecting smokers or never-smokers.

The last group of samples show evidence of defective DNA damage repair (DDR), including tumors with either mismatch repair (MMR; SBS6) or homologous recombination (HR; SBS3) defect signatures. We sought to identify the drivers of these signatures by identifying somatic or germline variants belonging to known genes in these genome integrity pathways, but we did not find any definitive germline driver mutations in these samples (Supplemental Figure 6C). Analysis of germline variants did reveal 4 samples with heterozygous pathogenic germline mutations in *MUTYH* (Supplemental Figure 6D). However, one of these samples had a dominant HR signature rather than the defective base excision repair signature expected from *MUTYH* deficiency. Further investigation is warranted to identify the underlying cause of DNA damage signatures in never-smokers and to determine if these mutations contribute to tumor initiation and therapeutic response. However, it should be noted that these signatures have a fairly flat profile and, with a limited number of mutations present in exome sequencing, their detection is inherently difficult.

### The mutation spectrum of KRAS and EGFR affects therapeutic options in never- and light smokers.

**Figure 3:**
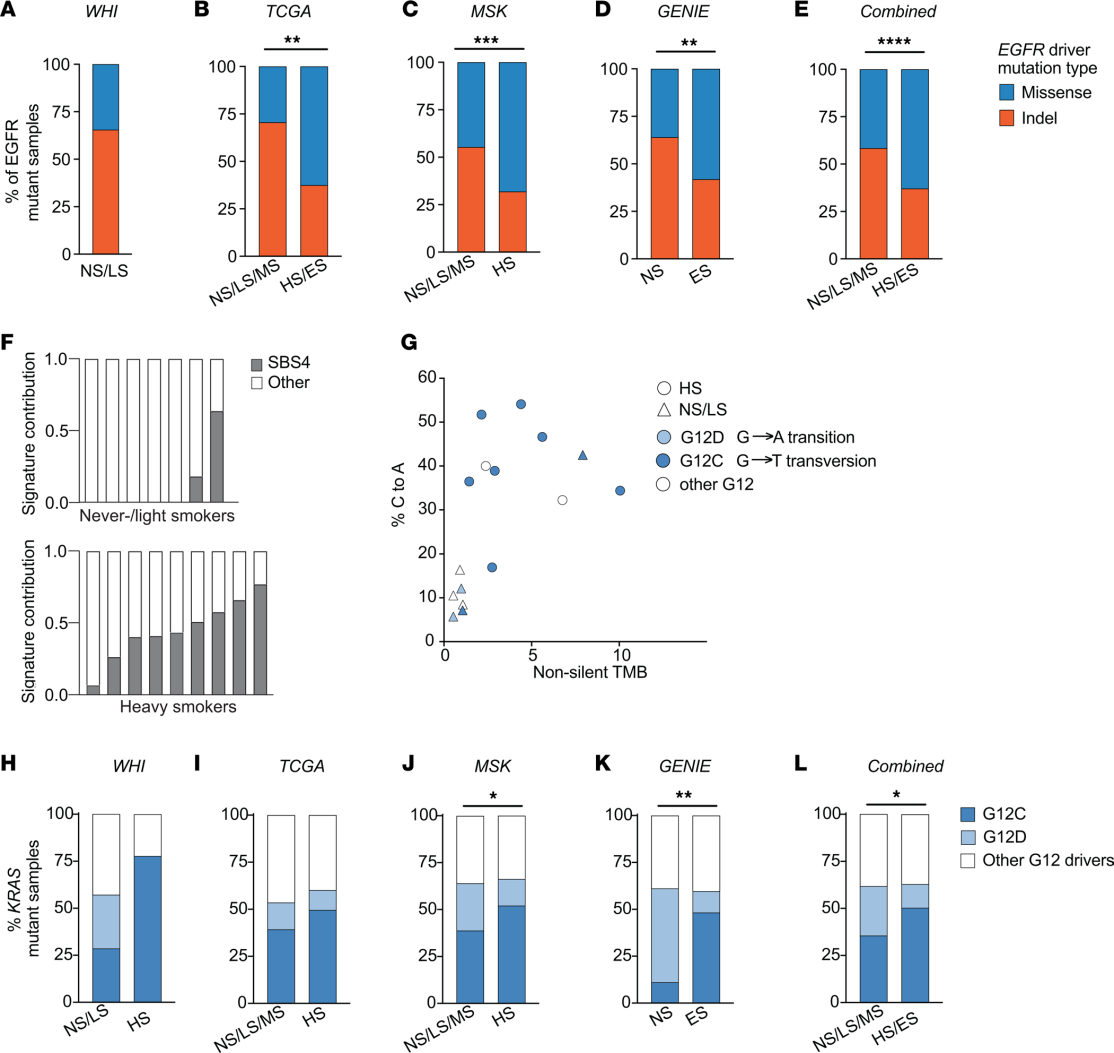
Enrichment of *EGFR* indel and specific *KRAS* variants in never-/light smokers. (**A**–**E**) Percent of *EGFR* driver mutations consisting of either indel or missense variants in WHI (**A**), TCGA (**B**), MSK (**C**), and GENIE (**D**) cohorts, or all external cohorts combined (**E**). (**F**) SBS4/Tobacco smoke signature contribution to the total mutational signature spectrum of samples with *KRAS* mutations. The contribution of SBS4 (gray bars) is the normalized contribution relative to the contribution of all other signatures (white bars). (**G**) Scatter plot of nonsilent tumor mutational burden (TMB) and percent C to A transversions in *KRAS-*mutant samples. (**H**–**L**) Percent *KRAS* G12 mutant samples with either G12C (dark blue), G12D (light blue), or other G12 drivers (white) in WHI (**H**), TCGA (**I**), MSK (**J**), or GENIE (**K**) cohorts or external cohorts combined (**L**). Statistical analysis was done using 2-tailed Fisher’s test. NS, never-smoker; LS, light smoker; MS, moderate smoker; HS, heavy smoker; ES, ever-smoker. ****P < 0.0001; ***P < 0.001; **P < 0.01; *P < 0.05 by 2-tailed Fisher’s exact test.

Given the substantial differences in the mutational signatures between smokers and never-/light smokers, we hypothesized that frequently mutated genes might also show differences in their mutational spectrums. Indeed, *EGFR* showed skewing of the type of mutations present depending on smoking status; 66% (19 of 29) of the *EGFR*-mutant samples in the never-/light smokers group were exon 19 deletions or exon 20 insertions, rather than missense variants such as L858R (Figure 3A) (54). Extending this analysis to the external MSK (Figure 3C) (29–32), TCGA (Figure 3B) (28), and GENIE (Figure 3D) cohorts confirmed a higher proportion of *EGFR* indel mutations in never-/light smokers compared with heavy smokers (Figure 3E). We also explored if age or sex influenced the occurrence of indel mutations in *EGFR*. There was no significant difference in *EGFR* indel mutation rate between males and females in each smoking subgroup (Supplemental Figure 7, A–D). However there was a modestly increased prevalence of *EGFR* indel mutations in individuals diagnosed with lung cancer before age 50, which reached statistical significance in the combined analysis (Supplemental Figure 7, E–H).

To determine if the high indel prevalence in the WHI cohort was limited to *EGFR* or was a general mutagenic property of these tumors, we analyzed the difference in the total number of indels by *EGFR* status (Supplemental Figure 7I). All tumors had a similar abundance of indel mutations regardless of their *EGFR* genotype in our cohort (indel versus WT, *P* = 0.129; indel versus missense, *P* = 0.5184; Mann-Whitney *U* test) (Supplemental Figure 7I), indicating there was no genome-wide indel-related signature altered in the tumors with *EGFR* indel mutations. Conversely, we explored whether the missense variants in *EGFR* in tumors from smokers were related to smoking-related mutagenesis. However, the nucleotide changes resulting in the L858R variant are not characteristic of smoking-induced mutagenesis (Supplemental Figure 7J) but rather to the clock-like mutational process.

*KRAS* mutations are frequent in tumors from smokers and, to a lesser extent, can be observed in tumors from never-smokers (22). We identified relatively prevalent mutation of *KRAS* in never-/light smokers (*n* = 7; 12.5%) in addition to the expected enrichment of *KRAS* mutations in heavy smokers (52.9%). To address if *KRAS* mutations occur in never-smokers due to passive smoke exposure, we queried the levels of the SBS4 tobacco signature in *KRAS*-mutant tumors. Heavy smokers with *KRAS* mutations uniformly exhibited an SBS4 tobacco smoking signature in their tumors (Figure 3F and Supplemental Table 7). In contrast, the SBS4 signature exposure was below the detection level in 5 of 7 tumors in the never-/light smoker group. These data demonstrate that *KRAS* mutations can occur in the absence of smoke exposure.

The predominant site for mutation in *KRAS* in lung cancer is glycine 12, and all *KRAS*-mutant tumors in our cohort were mutated at that site. However, the specific amino acid variant introduced differed between never-/light smokers and heavy smokers (Figure 3G). *KRAS* mutations in tumors from smokers were predominantly glycine to cysteine (G12C) variants (7 of 9 or 78%), whereas never-/light smokers had fewer G12C variants (2 of 7 or 28.5%) and a higher percentage of glycine to aspartic acid (G12D) variants (28%; Figure 3H). Extrapolating this observation to the MSK, TCGA, and GENIE studies, we see that G12C mutations were significantly enriched in heavy smokers compared with never-/light/moderate smokers (Figure 3, I–L). Sex and age did not affect the enrichment of G12C variants in either smoking subgroup (Supplemental Figure 7, K–R). Currently, G12C is the only clinically druggable *KRAS* variant (55, 56), so these differences in genotype prevalence affect patients’ access to the newly available *KRAS-*targeted therapies.

### The somatic copy number landscape is similar between never and heavy smokers.

**Figure 4:**
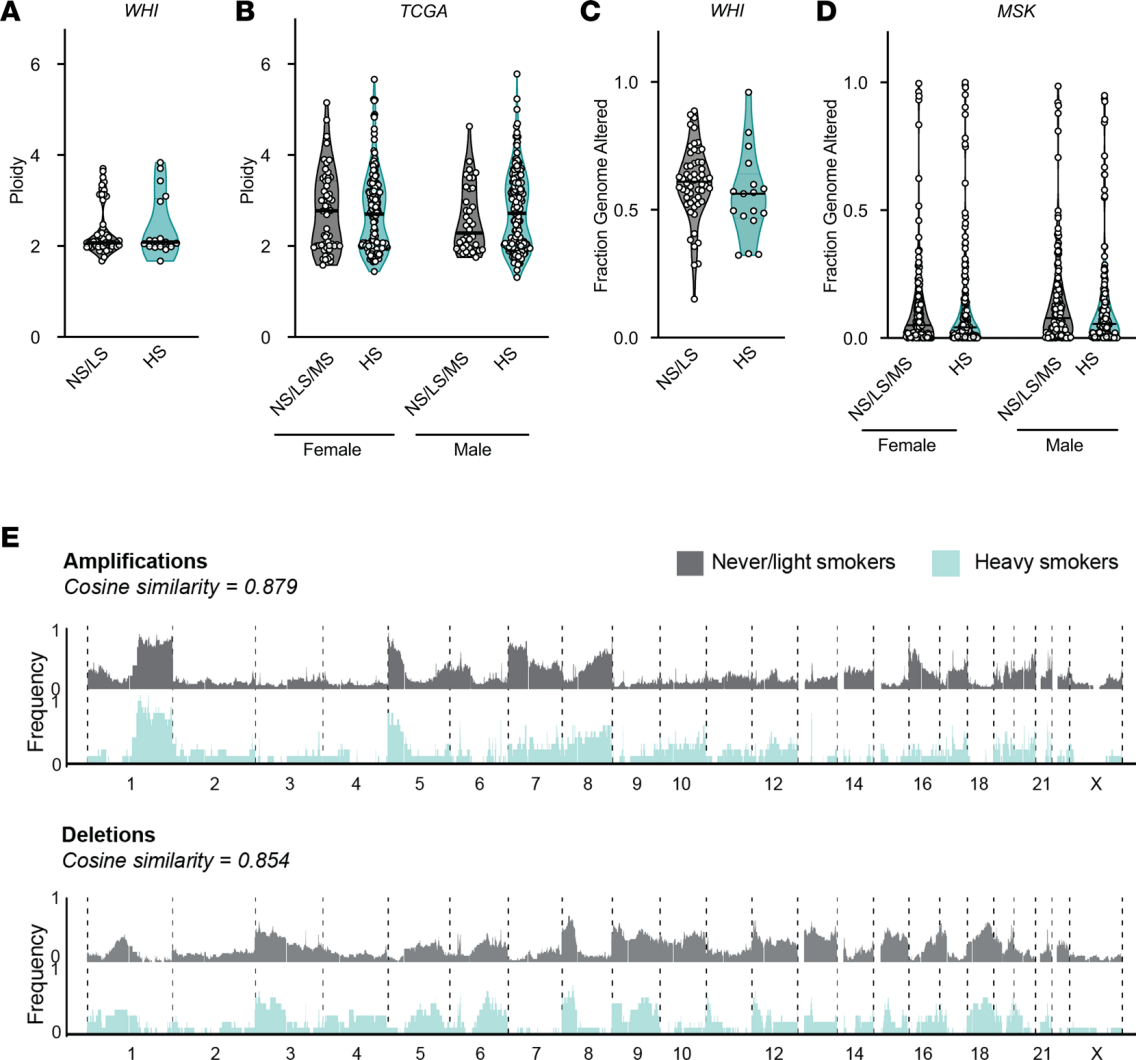
Somatic copy number changes do not differentiate tumors from never-/light smokers and heavy smokers. (**A** and **B**) Ploidy of tumors from the WHI cohort or TCGA cohort. (**C** and **D**) Fraction genome altered (FGA) of from the WHI cohort or MSK cohort. (**E**) Genome-wide frequency of amplifications and deletions in never-/light smokers (gray/top panels) and heavy smokers (blue/bottom panels) across all 23 chromosomes. Mann-Whitney *U* test was used to evaluate significant difference in ploidy and FGA between groups. Cosine similarity was calculated between both smoking groups for amplifications (top) and deletions (bottom).

SCNAs are distinctive features of cancer genomes (4, 57–59). SCNAs include focal amplifications and deletions, chromosome arm-level events, aneuploidy, and whole genome doubling (60, 61). Whereas the role of smoking on SNV mutagenesis is well documented, the effect of smoking on aneuploidy is not well understood. Ploidy is frequently altered in cancer due to underlying errors in cell division (62). These ploidy changes are often due to whole genome duplications, sometimes followed by secondary gains or losses of full or partial sets of chromosomes. Despite the extensive differences in tumor somatic mutation patterns between heavy and never-/light smokers, we surprisingly found no significant difference in ploidy between these groups both in our cohort (WHI cohort Figure 4A and Supplemental Table 8; *P* = 0.86; Mann-Whitney *U* test) as well as external cohorts (Figure 4B and Supplemental Figure 8, A–C). Furthermore, ploidy did not correlate with pack-years of smoke exposure in the WHI cohort (Spearman *r* = –0.06, *P* = 0.612) or in the TCGA cohort (Spearman *r* = 0.060, *P* = 0.154) (Supplemental Figure 8, D–H). Whole genome duplication was not affected by sex or age (Supplemental Figure 8, A–C and E–H).

We next examined the difference in the fraction genome altered (FGA) between never-/light smokers and heavy smokers. Unlike ploidy, FGA describes the overall breadth of the genome altered rather than the amplitude of that alteration across the whole genome. There was no significant difference in the FGA between both never-/light and heavy smokers in the WHI cohort (*P* > 0.05) (Figure 4C) or in the MSKcohort (Figure 4D) nor was there a positive correlation with pack-year smoke exposure (Spearman’s correlation *r* = –0.19, *P* = 0.10) in either the WHI or MSK cohort (Supplemental Figure 9, A–C). Age also did not influence FGA (Supplemental Figure 9D). Consistent with these observations, the total number of amplifications or deletions did not differ by smoke exposure (Supplemental Figure 9E). Last, we compared the pattern of recurrent amplifications and deletions across the genome between never-/light and heavy smokers (Figure 4C). Overall, the frequency of alterations at specific chromosomal locations showed broadly similar patterns. Therefore, smoking does not appear to influence the quantitative and qualitative metrics of genome-wide copy number patterns in lung adenocarcinoma.

### Arm-level copy number alterations cluster tumors independently of smoking status.

**Figure 5:**
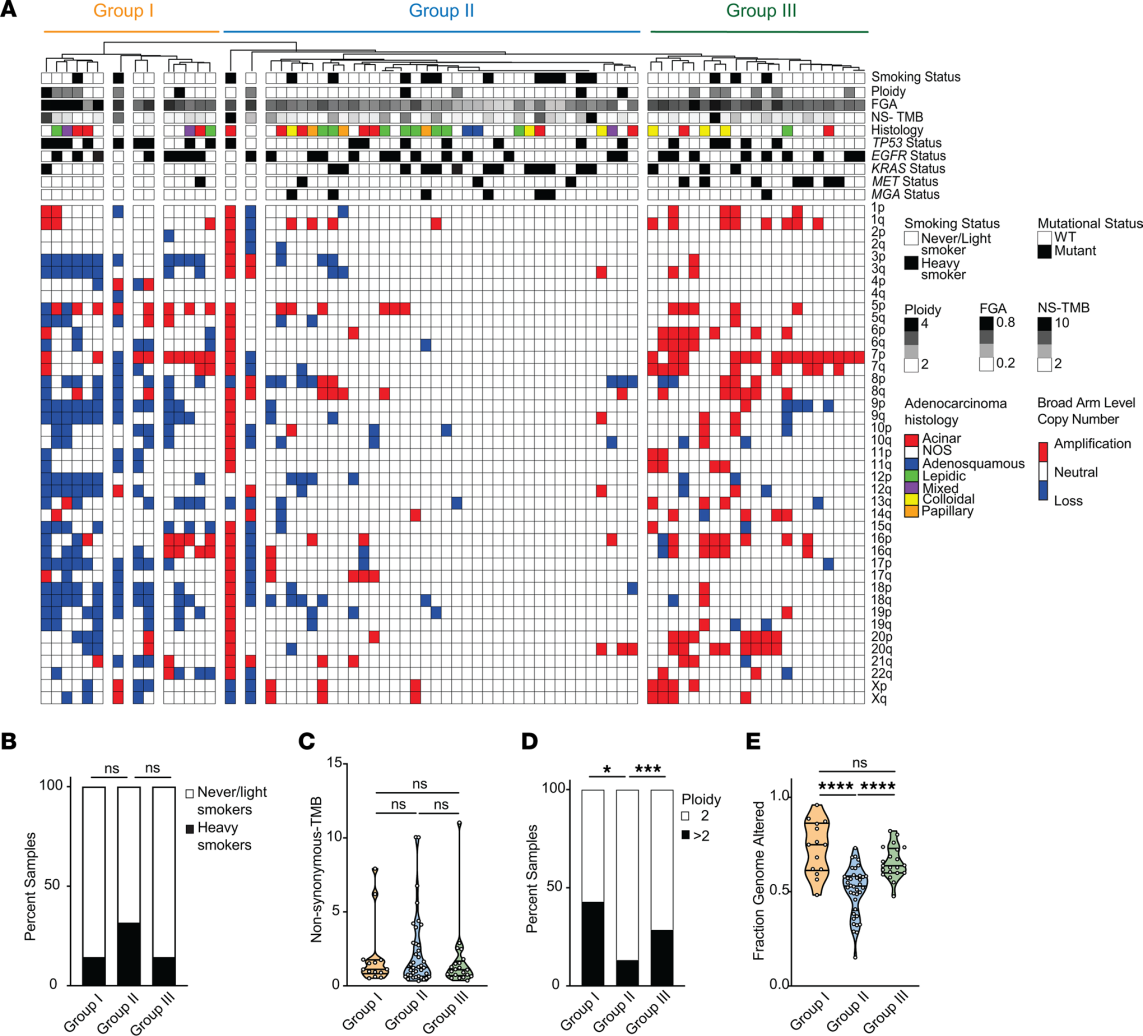
Arm-level copy number alterations identify tumor subtypes unrelated to smoke exposure. (**A**) Heatmap of unsupervised clustering of arm-level copy number alterations in the WHI cohort using Ward’s minimum variance method for both samples and signatures. The clustering is based on binarized arm-level calls from GISTIC 2.0. Samples were grouped into 3 groups based on broad clusters and copy number patterns. (**B**) Stacked bar graph showing the percent of never-/smokers and heavy smokers in each copy number group. Fisher’s exact test was used to compare the number of NS/LS in each copy number group compared with the other. (**C**) Nonsilent TMB in samples split by arm-level copy number group. Mann-Whitney *U* test. Group I, orange; Group II, blue; and Group III, green. (**D**) Stacked bar graph showing percent samples in each group with ploidy 2 or ploidy greater than 2. Black bars indicate a ploidy estimate greater than 2, and white bars indicate a ploidy estimate of 2. Fisher’s exact test was used to compare enrichment of ploidy > 2 in each copy number group. ***P < 0.001, *P < 0.05. (**E**) Fraction genome altered in samples split by arm-level copy number group. One-way ANOVA/Tukey was used to compare significance between the different groups. **** *P* < 0.0001.

SCNA burden is associated with poor overall survival and is being considered as a potential biomarker of recurrence and therapy (59, 63–65). One of the largest whole-genome studies of lung cancer from never-smokers showed that tumors from never-smokers contain frequent arm-level copy number alterations, and these can be used to cluster tumors into distinct groups with increasing aneuploidy (12). We performed unsupervised clustering of arm-level copy number events in tumors from WHI (Supplemental Table 9) and recapitulated 3 copy number groups similar to those previously described: Group I (*n* = 14), Group II (*n* = 38), and Group III (*n* = 21) (Figure 5A). Group I was enriched for arm-level deletion events of 3p, 9p, and 17p (60). Group II included 52% (*n* = 38) of all samples in the cohort with very few arm-level events. Group III showed significant enrichment of amplifications of 7p, 7q, 6p, and 20p compared with the other 2 groups. However, we did not observe amplification of 1q or 5p in this group, unlike the previous study (12).

Interestingly, smoking history did not appear to influence tumor clustering (Figure 5B). Consistently, there was no significant difference in nonsilent TMB between the 3 groups that would suggest a role for smoking in the tumor clustering (Figure 5C; Mann-Whitney, *P* > 0.05). *TP5*3 mutations were enriched in samples in Group I (Fisher’s exact test, *P* = 0.0183) and *KRAS*-mutant samples in Group II (Fisher’s exact test, *P* = 0.0492). Whole genome duplication (or ploidy > 2) was significantly higher in both Group I and III compared with Group II (Fisher’s exact tests *P* < 0.05) (Figure 5D), with FGA also increased in Group I and III (unpaired, 2-tailed *t* test, *P* < 0.05) (Figure 5E). Overall, we found that these aneuploidy-based clusters were independent of the patient’s smoking history and instead represent a general feature of all lung adenocarcinomas.

## Discussion

In this study, we present the genomic landscape of lung adenocarcinoma in female never- and light smokers from the WHI. Our work complements recent proteogenomic and genomic analyses (12, 13, 16, 53, 66), but specifically focuses on older women, the demographic that accounts for the largest fraction of lung cancer cases not related to smoking. We confirm previously discovered genomic differences between never-/light and heavy smokers such as the enrichment of *EGFR* mutations in never-smokers. We also uncover unique features of the genomic landscape that have not been previously described, such as the enrichment of *MGA* mutations in heavy smokers. Our data also point to interesting age-related differences in the pathogenesis of lung cancer such as the increased prevalence of RTK fusions and *EGFR* indel mutations in lung cancer patients younger than 50.

While somatic SNV and indel mutational patterns are distinct between never-/light smokers and heavy smokers, surprisingly, we found no association between smoking status and aneuploidy. Therefore, the recently described copy number subtypes of lung tumors in never-smokers (12) are likely to represent a general-feature lung cancer not unique to tumors from smokers or never-smokers.

Despite the similar patterns of aneuploidy, tumors from never-smokers and smokers show vast differences in somatic mutation landscape with important implications for therapeutic options and efficacy. First, somatic TMB, an indicator of neoantigen burden, is suggested to predict to a certain extent immunotherapy response (31, 67). Thus the low TMB of tumors from never-smokers may partially contribute to their immunologically “cold” phenotype (68). Second, we show that the mutational spectrum of variants in important clinical targets *EGFR* and *KRAS* are significantly different in tumors from never-/light and heavy smokers. *EGFR* indel mutations are enriched in tumors from never-/light smokers, while missense mutations are more common in heavy smokers. The reason for this difference is yet unclear, but it may be related to differences in DDR mechanisms or other mechanisms of mutagenesis and could be an important clue to the etiology of *EGFR*-mutant lung cancer.

Interestingly, we also found *KRAS* mutations in tumors with no tobacco smoke exposure, but the targetable G12C mutation is much less prevalent in these tumors. Thus, we suggest that never-/light smokers with *KRAS*-mutant disease are a population with particular unmet medical needs; these tumors naturally lack other targetable biomarkers such as *EGFR* mutations, do not contain a targetable *KRAS* variant, and are unlikely to respond to immunotherapy due to low mutational burden. Fortunately, the development of additional *KRAS* inhibitors is underway to partially address this challenge.

Exome-wide mutational signatures provide a clear view of mutational processes at work, such as the distinctive SBS4 or “transversion-high” signature observed in tumors from smokers (18, 28). We reasoned that focused analysis of tumors from never-/light smokers might reveal clues to mutagenic processes and the etiology of lung cancer in the absence of smoking. However, we observed no signatures that were uniquely abundant in tumors from never-/light smokers. It is important to note here that the NMF-based signature analysis is highly sensitive to input parameters, and some signatures are hard to determine confidently from exome sequencing data. To address these challenges, we restricted our analysis to signatures previously reported in literature. It is possible that low frequency or unique mutational processes would be missed with this approach. Our results show that age-related clock mutagenesis and APOBEC mutagenesis are clearly operative in tumors from never-smokers but contribute to a similar mutational burden as that seen in tumors from heavy smokers. Thus, we cannot attribute the cancer development in never-/light smokers to any unique environmental or endogenous mutational process at this time. As the number of lung cancer cases in never-smokers appears to be increasing (9), it is crucial to continue to better understand the molecular mechanisms of tumor development in never-smokers to develop effective prevention and treatment strategies.

## Methods

### Sex as a biological variable.

Our study focused on lung adenocarcinoma samples from female participants. This decision was grounded in the observation that lung cancer in never-smokers is found to occur more frequently in women than in men, even after adjusting for smoking behavior. This pattern suggests that biological sex may influence the incidence of lung cancer among never-smokers, driving the design of this study. Additionally, we compared genetic findings between males and females using external cohorts and discussed any significant differences that could be attributed to biological sex.

### Sample inclusion criteria.

The participants of the current study were all postmenopausal women retrospectively selected from the WHI cohort. Initial selection criteria for participants included a lung adenocarcinoma diagnosis and smoking history of less than 100 lifetime cigarettes (never-smokers), less than 5 pack-years (light smokers), or greater than 20 pack-years (heavy smokers). All cohort participants were matched for cancer stage, diagnosis year, and tumor purity. Patient characteristics are provided in Supplemental Table 1.

### Pathology review and tissue samples.

H&E slides from formalin-fixed, paraffin-embedded (FFPE) tumors were requested from the WHI for the participants fulfilling the above selection criteria. All tissue samples were generated from either diagnostic surgery/lobectomy/segmentectomy/resection procedures. These sections were reviewed by a pathologist for histological confirmation of the lung adenocarcinoma diagnosis and tumor content, and purity was checked for sufficiency for sequencing. Tumor cells were identified by the pathologist and macrodissected to enrich for tumor purity. A total of 73 participants with sufficient tumor availability for sequencing was included in the present study (Supplemental Tables 1 and 2). While the tumor source for this study was derived from FFPE tissue, each sample had a matched normal/control, derived from fresh frozen peripheral blood. To control for FFPE-induced changes, 9 tumor-adjacent normal samples from participants in the study were also sequenced.

### Genomic DNA isolation for sequencing.

Tumor and tumor-adjacent normal FFPE tissues were macrodissected, guided by pathological review of sections. Genomic DNA was isolated using the QIAGEN QIAmp DNA FFPE kit (catalog 56404) with some modifications. DNA from matched normal was derived from the buffy coats of prepared blood samples. A salting-out method was used to purify the genomic DNA. RBCs were first lysed and washed out, and then the WBC nuclei underwent lysis. Cellular proteins were precipitated and removed, followed by DNA precipitation.

### Custom WES and preanalysis processing.

Custom WES was performed using DNA derived from tumor/normal FFPE tissue and fresh frozen peripheral blood. In total, 250 ng of FFPE-derived DNA and 150 ng of fresh frozen blood DNA were used for library construction. Normalized genomic DNA was fragmented to an average size of 250 bp, and size-selected DNA was ligated to adapters. Libraries were pooled and sequenced to quantify library yields. Pooled libraries were then captured using a custom bait set (27), which targets the entire exome and intronic regions known to have structural rearrangements commonly occurring in cancer. This custom bait set was a combination of the Agilent Exome v5 bait set and a custom bait set targeted at regions of known structural rearrangements known as “POPv3.1_SV_ONLY” (design ID 319145; OncoPanel [POPv3.1]). The genes and regions targeted by the SV bait set can be found in Supplemental Figure 4 of the publication cited (27). This SV bait set targeted 60 genes and covered 191 regions, including intronic regions. Hybrid captures were then sequenced on NovaSeq flow cells. Sequencing metrics are provided in Supplemental Table 2. Read pairs were aligned to the hg19 reference sequence using the Burrows-Wheeler Aligner (69), and data were sorted and duplicate-marked using Picard tools. The alignments were further refined using the Genome Analysis Toolkit (GATK) (70, 71) for localized realignment around indel sites, and recalibration of quality scores was also performed. The complete analysis pipeline for alignment can be found at https://github.com/FredHutch/tg-wdl-LILAC-workflow (commit ID: 1edb6ebb3e8417553ef12a41e18911065f96b19f).

Tumor and matched normal DNA pairing were unknown prior to sequencing; therefore, a fingerprinting analysis was performed using 44 polymorphic loci to identify the pairing. Picard Tools GenotypeConcordance was used to calculate the concordance that a given test sample matches the sample being considered. This was performed on all pairwise combinations of samples in the cohort. The output of the pairwise comparisons was then mapped to a concordance matrix, where concordance values above 4 SDs of the median concordance value for the cohort indicated a high likelihood that the samples match. Potential matches are manually reviewed and confirmed for accuracy from the WHI.

### Whole genome sequencing and preprocessing.

Genomic DNA was quantified using Life Technologies Invitrogen Qubit 2.0 Fluorometer (Thermo Fisher Scientific) and fragmented using a Covaris LE220 ultrasonicator (Covaris) targeting 400 bp. Sequencing libraries were prepared using 100 ng fragmented FFPE DNA using the IDT xGen cfDNA & FFPE DNA Library Prep v2 MC and xGen Indexing Primers (Integrated DNA Technologies). Library quantification was performed using Life Technologies Invitrogen Qubit 2.0 Fluorometer and size distribution validated using an Agilent 4200 TapeStation (Agilent Technologies). Individual libraries were pooled (11-plex) at equimolar concentrations and sequenced on an Illumina NovaSeq 6000 using an S4-300 flow cell employing a paired-end, 150 bp read length sequencing configuration.

Basecalling and demultiplexing were performed with Illumina bcl2fastq v2.20. Demultiplexing was configured to trim the unique 8 bp UMI sequences from each read in a pair, preserving them in the read names in the resulting FastQ files. Reads were then trimmed with cutadapt 4.1 (72) and aligned to the hg19 human genome reference using BWA MEM 0.7.17 (73). A custom script was used to postprocess the resulting alignments by adding UMI sequences as a tag (“RX”) for each alignment. This tag was used to perform UMI-aware deduplication with Picard MarkDuplicates 2.25.1 (Broad 2019; https://broadinstitute.github.io/picard/; commit ID: 044bcdaf77860488c9e3688e9b7b967073deffef).

### Mutation calling.

To define a high-confidence map of somatic SNVs and indels, we called mutations using a custom mutation-calling strategy involving 3 somatic callers: MuTect2, Strelka, and SvABA. Analysis-ready BAM files were analyzed using GATK-MuTect 2 (version 4.1.4.0) run with the FFPE bias filter and a panel of normals (PoN) including the FFPE normal samples to help exclude potential FFPE artifacts (74). BAM files were also processed through Strelka (v2.9.10) (75) with Manta (v1.6.0) (76), and BAMs were evaluated using the SvABA algorithm (45). The complete analysis pipeline for alignment and somatic SNV and indel calling using MuTect2 and Strelka can be found at https://github.com/FredHutch/tg-wdl-LILAC-workflow (commit ID: 1edb6ebb3e8417553ef12a41e18911065f96b19f). SNV calls that passed both MuTect2 and Strelka were included in the final call set. Indel calls that passed at least 2 of the 3 callers were included in the final call set. Finally, those SNVs and indels with variant allele frequencies (VAF) greater than 10% in gnomAD or ExAC databases were filtered out to generate the final call set for further analysis. Significant SNV and indel mutations were identified using the MutSig2CV (74, 77) algorithm. Nonsilent tumor-mutational burden was derived from MutSig2CV output. Percent C to A transversions were calculated using the SNV data from the final call set.

### SV analysis by SvABA.

Somatic SVs from each tumor-normal pair sequenced by the custom WES and the tumor-only setting sequenced by whole genome sequencing were identified using SvABA (45). SvABA analysis was performed using default tumor-normal paired settings for WES and tumor-only settings for the WGS. Events that passed the default SvABA filter were included in the final analysis. All the SV calls were converted to VCF format, and the resulting files were annotated with gene information from GENCODE HG19 version. Additionally SV calls that were filtered out were manually evaluated for call-rescue. None of the filtered-out calls were rescued, since they lacked sufficient evidence to be confident calls. All calls were reviewed for translocations in *ALK*, *RET*, and *ROS1*. Furthermore, calls were surveyed for known functional translocations in cancer.

For the SV calls generated from WGS, Circos plots were created using Rcircos (78) and by selecting SVs in protein-coding regions (at least 1 breakpoint) and if they are greater than 10 kb length. This filtering was done on the VCF files of PASS calls from SvABA. For the Circos plots, all interchromosomal events are shown in blue and intrachromosomal events are in red. A list of known gene fusions involving *ROS1*, *ALK*, and *RET* genes was created using COSMIC and was manually curated from peer-reviewed literature. For each of these 75 fusions, we investigated the same breakpoints in the 11 WGS samples and visualized them using SamPlot (79) and IGV (80).

### Fusion detection and mutational analysis from targeted resequencing by AmpliSeq.

To perform deeper targeted sequencing, we utilized Illumina’s AmpliSeq Focus Panel to identify missed mutational calls in otherwise oncogene-negative samples and fusions. For the DNA sequencing panel, genomic DNA extracted for custom WES was used as input for the targeted sequencing. RNA was extracted from FFPE slides/curls using the QIAGEN RNeasy FFPE Kit (catalog 73504).

All amplicon samples were sequenced on an Illumina MiSeq using a paired-end 150 bp read configuration. Raw data were collected using Illumina Real Time Analysis (RTA) software 1.18.54.4, with subsequent base calling and demultiplexing performed with bcl2fastq 2.20 (https://support.illumina.com/sequencing/sequencing_software/bcl2fastq-conversion-software.html). All samples were sequenced to an average depth of 300,000 read pairs each.

For DNA amplicon analysis, AmpliSeq samples were processed with the Illumina DNA Amplicon Workflow v3.0.0.14, which internally uses BWA MEM 0.7.9a (69) to align paired reads to the GRCh37/hg19 human reference, followed by variant calling with Pisces in targeted amplicon region. Resulting DNA variants were annotated with GATK (v4.1.8.1) Funcotator along with funcotator_dataSources.v1.6.20190124s (71).

For RNA fusion analysis, AmpliSeq samples were processed with the Illumina RNA Amplicon Workflow 3.0.0.26. This analysis method also uses BWA MEM internally to align reads to targeted regions and fusions, followed by proprietary methods to call gene fusions and exon variants.

### Cell-line and PDX models.

The PDX was generated in-house from a patient (unpublished), and H2228 cell line was received as a gift from William Hahn, Broad Institute (Cambridge, Massachusetts, USA).

### Analysis of external data sets.

External genomic data sets were used to validate the findings made in the present study. All data referred to as TCGA cohort was derived from ref. 28; MSK (29–32) data was derived from cBioportal (81–83) lung adenocarcinoma studies. For analysis associated with GENIE, data was derived from NSCLC cohort v2.0-public (GENIE 2022) GENIE Release 11.1-public (accessed 3/12/2024), for patients with lung adenocarcinoma from Dana-Farber Cancer Institute (DFCI), Vanderbilt-Ingram Cancer Center (VICC), or Princess Margaret Cancer Centre - University Health Network (UHN) (33–35, 83).

The data downloaded from cBioportal (81, 83) were processed to annotate variants for *MGA*, *MET*, *EGFR*, and *KRAS*. For analysis of MGA mutations, samples where *MGA* was not profiled were excluded. In *MET* mutation analysis, oncogenic mutations, including *MET* ex14 skipping, were considered mutant, while uncertain significance mutations were labeled VUS. *EGFR* mutations were classified as mutant if clearly designated as drivers (missense or indel).

For fusion analysis data from MSK, OncoSG, TCGA, Broad Institute, and NCI SHERLOCK (4, 5, 12, 30, 32, 46) data were utilized. Never-/light/moderate smokers were from individuals with a less than 20 pack-year history or tagged as former-light smokers in these studies. For heavy smokers were those individuals with a 20 or greater pack-year history or current or former heavy smoker tag in the studies. Ever-smokers included individuals who were tagged as ever, current, or current reformed smokers. Only those samples were included where *ALK*, *RET*, and *ROS1* were profiled. For analysis involving age at diagnosis and stage, both male and female were combined.

### Mutational signature analysis.

Mutational signature analysis was performed using the R-based package Sigminer and the final variant call file generated for SNVs and indels. Sigminer utilizes a nonnegative matrix factorization–based (NMF-based) approach for mutational signature determination (48, 84–94). Signature extraction was performed for all samples and then matched to known COSMIC v3.1 signatures. Signature matches were then compared with those signatures reported in literature associated with lung adenocarcinoma. A final signature refitting was then performed for our samples limited to these reported signatures to form the final mutational signature exposure matrix. Unsupervised clustering was then performed on these mutational signature exposures using the Ward.D.2 minimum variance method, using Euclidean distance.

### Copy number analysis.

To determine the copy number alterations, we utilized the TITAN pipeline (https://github.com/gavinha/TitanCNA; commit ID: 2b55d94fca707826496bf46e4aadde14447cf703) (95, 96). Corrected read counts were determined in nonoverlapping windows of 50 kb that also overlapped the bait intervals by at least 1 bp. We also utilized the FFPE “normal” samples to normalize any FFPE-induced copy number changes. All TITAN calls were then subject to manual curation to verify the optimal ploidy solution. Curated optimal solutions are shown in Supplemental Table 8. Copy number data from TITAN were then put into GISTIC 2.0 to determine regions with significant copy number alterations. FGA was calculated by dividing the sum of all amplified/deleted segments by the total number of segments for each patient. Arm-level copy number was determined from GISTIC output, and unsupervised clustering was performed using Ward’s minimum variance method.

### Statistics.

Statistical tests were performed using R or GraphPad Prism 10 software. For continuous variables, first normality of the data was tested using the Shapiro-Wilk test. For comparisons involving 2 groups if the data were normal, a 2-tailed *t* test was performed; if not, a Mann-Whitney *U* test was performed. *P* values were then annotated using stars. For comparisons involving 3 or more groups, if all groups passed the normality test, a 1-way ANOVA was performed, followed by the Tukey’s test for significance. If any group failed the normality test, a Kruskal-Wallis test was performed, followed by Dunn’s post hoc test with Benjamini-Hochberg correction, if the Kruskal-Wallis test was significant. Simple linear regression was used to assess the linear relationship between 2 variables. The resulting *P* value indicated the probability of observing the data under the null hypothesis of no relationship between the variables. *P* < 0.05 was considered statistically significant. Categorical variables were compared using a 2-tailed Fisher’s exact test, with *P* < 0.05 considered statistically significant. Hierarchical clustering was performed using Ward’s minimum variance method, and the hierarchical clustering dendrogram was constructed based on Euclidean distances between data points. The cosine similarity measure was utilized to quantify the similarity between pairs of vectors in the copy number data set. This measure calculates the cosine of the angle between 2 vectors. A higher cosine similarity score suggests greater similarity, while a lower score indicates dissimilarity.

### Study approval.

All samples were obtained from patients after approval from the Fred Hutch Cancer Center IRB (no. 8667, protocol no. RG1001808) and appropriate informed consent from participants. Patient data including sample identifiers, patient identifiers, and metadata identifiers were d-identified from the authors. No identifiable private information was generated in this analysis. The WHI consent group to which each sample belongs is identified in Supplemental Table 1.

### Data availability.

Somatic mutation data (SNV and Indels specifically) can be found in Supplemental Tables 3 and 4. Protected genomic data for all samples with appropriate consent will be submitted to dbGaP under substudy accession phs003433 under the parent WHI study no. phs000200 for controlled-access use. Values for all data points in graphs are reported in the Supporting Data Values file.

## Author contributions

Conceptualization was contributed by GLA and AHB. Data acquisition was contributed by SM, AN, NAP, CS, ART, GLA, and AHB. Methodology was contributed by SM, AP, MK, ACHH, AN, NAP, FW, MPF, CS, ART, and GH. Analysis was contributed by SM, AP, PI, KML, FW, MPF, MP, and AHB. Writing of the original draft was contributed by SM, GH, and AHB. Review and editing were contributed by all authors. Funding acquisition was contributed by SM, AHB, GLA, and GH.

## Supplementary Material

Supplemental data

Supplemental tables 1-9

Supporting data values

## Figures and Tables

**Table 1:**
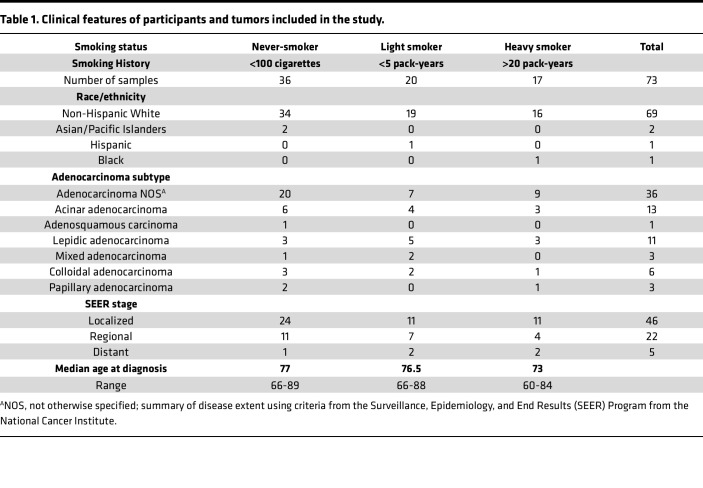
Clinical features of participants and tumors included in the study.

## Version 1 07/25/2024

In-Press Preview

## Version 2 09/10/2024

Electronic publication
