# Supplementary material for: The genomic landscape of lung cancer in never-smokers from the Women’s Health Initiative
Source: JCI Insight. 2024 Jul 25;9(17):e174643. doi: 10.1172/jci.insight.174643 (PMC11385083; doi:10.1172/jci.insight.174643)
Supplement: Supplemental data [file jciinsight-9-174643-s251.pdf]

FIGURE S1

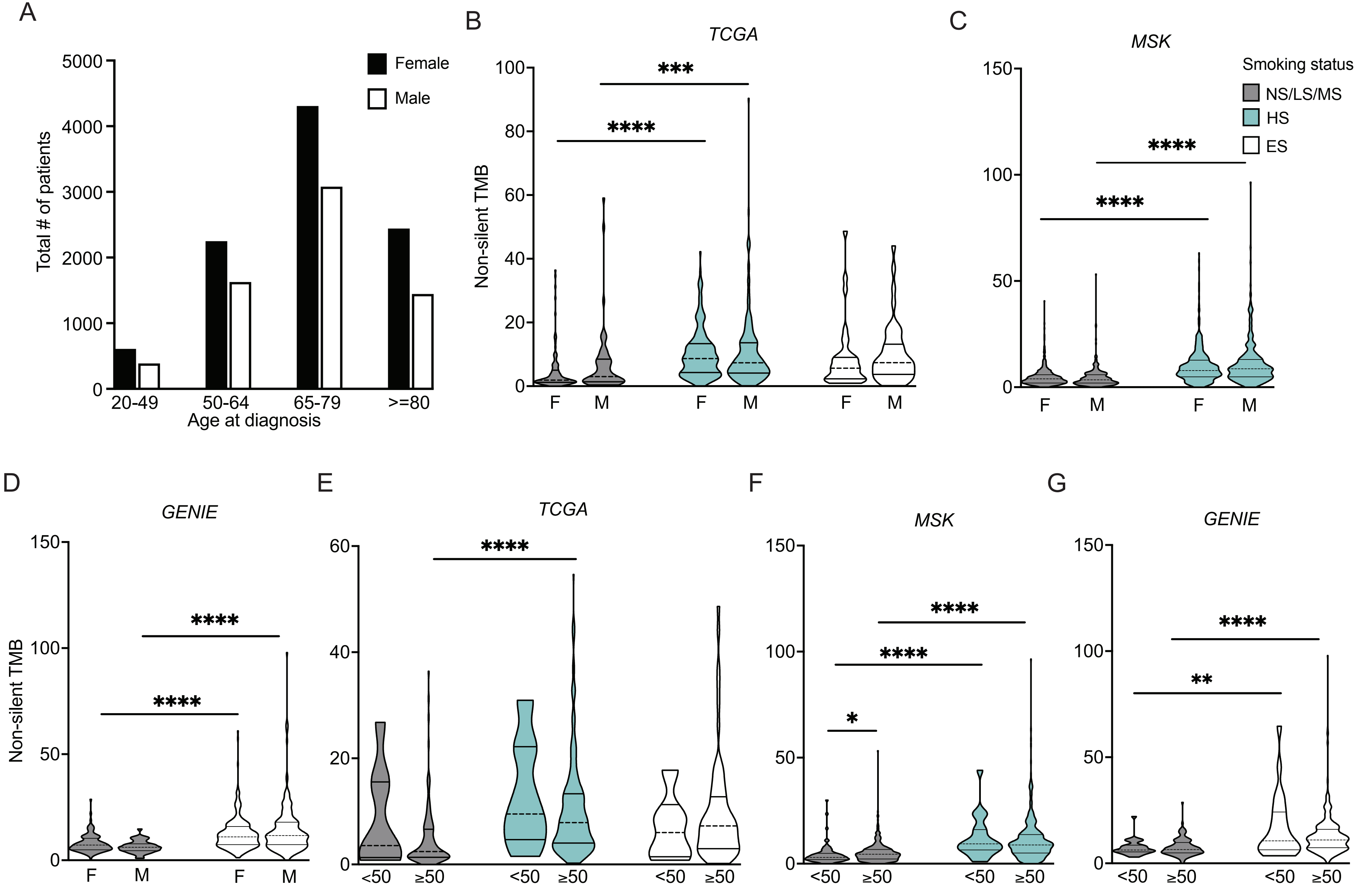

A
Female
Male
Total # of patients
5000
4000
3000
2000
1000
0
20-49
50-64
65-79
>=80
Age at diagnosis
B
TCGA
Non-silent TMB
***
****
F
M
C
MSK
Smoking status
NS/LS/MS
HS
ES
****
D
GENIE
E
TCGA
<50
≥50
F
MSK
*
G
GENIE
**

Figure S1. (A) Number of female and male never-smokers diagnosed with lung cancer stratified by age at diagnosis from Siegel *et al.*, 2021. **(B-D)** Non-silent TMB in males and females from the TCGA (B), MSK (C) and GENIE (D) cohorts. **(E-G)** Non-silent TMB in by age at diagnosis less than 50 years versus those diagnosed at 50 or more years from the TCGA (E), MSK (F) and GENIE (G) cohorts respectively. Kruskal-Wallis/Dunns test was used for comparison of TMB between smoking groups. p-values: * $p<0.05$ ** $p<0.01$. NS (Never-smoker), LS (Light smoker), MS (Moderate smoker), HS (Heavy smoker), ES (Ever-smoker), F (female), M (male).

FIGURE S2

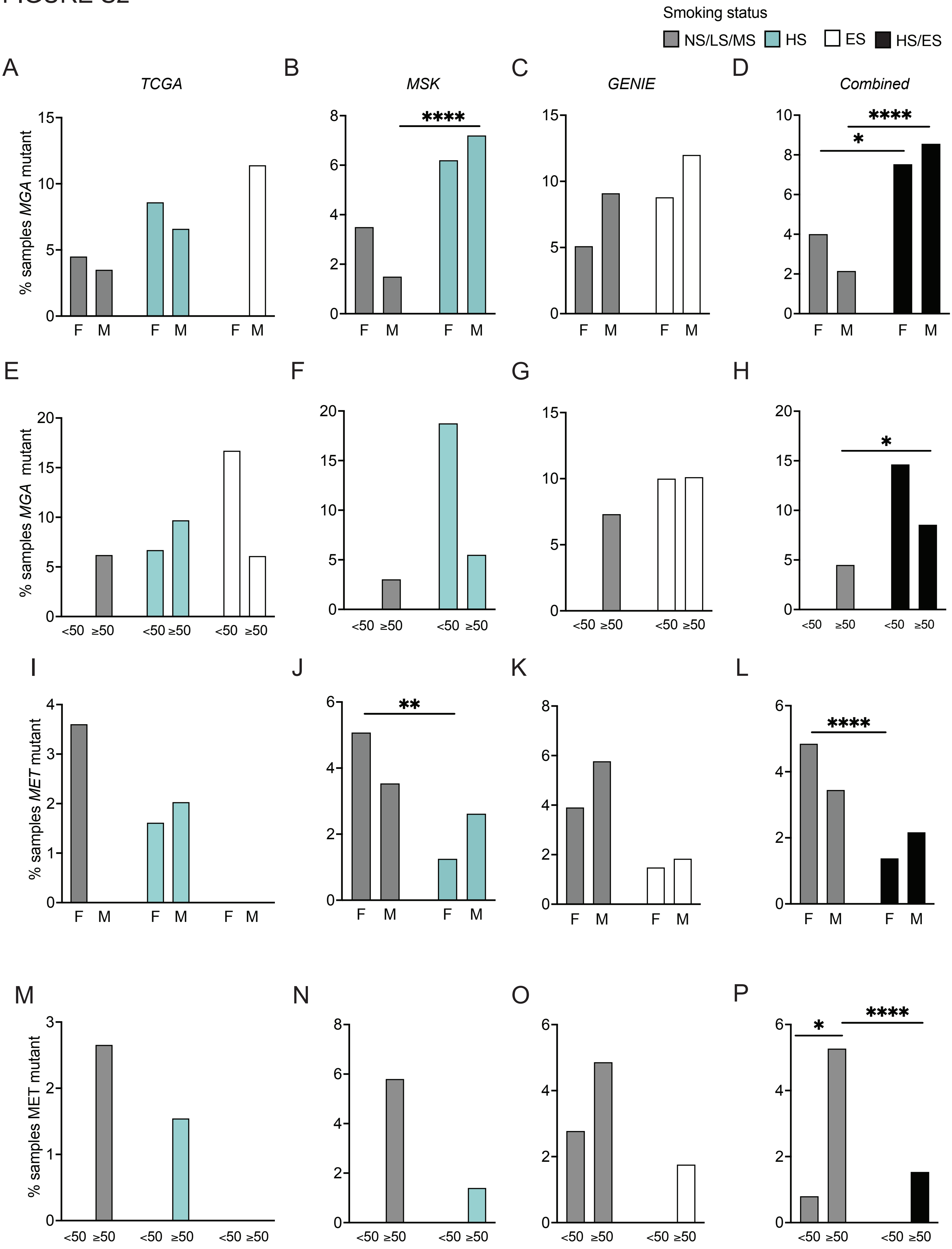
Smoking status
NS/LS/MS
HS
ES
HS/ES
A
B
C
D
TCGA
MSK
GENIE
Combined
% samples MGA mutant
F
M
****
*
E
F
G
H
<50
≥50
I
J
K
L
% samples MET mutant
**
M
N
O
P
% samples MET mutant

Figure S2. (A-D) Percent samples with *MGA* mutations split by smoking status and sex in the TCGA (A), MSK (B), GENIE (C) cohorts and the three cohorts combined (D). Fisher's exact test was used to compare the enrichment of *MGA* mutations between smoking groups. **(E-H)** Percent samples with *MGA* mutations split by age at diagnosis in the TCGA (E), MSK (F), GENIE (G) cohorts and the three cohorts combined(H). Fisher's exact test was used to compare the enrichment of *MGA* mutations between the age of diagnosis groups. **(I-L)** Percent samples with *MET* mutations split by smoking status in the TCGA(I), MSK (J), GENIE(K) cohorts and the three cohorts combined(L). Fisher's exact test was used to compare the enrichment of *MET* mutations between smoking groups. **(M-P)** Percent samples with *MET* mutations split by age at diagnosis in the TCGA(M), MSK (N), GENIE(O) cohorts and the three cohorts combined(P). Fisher's exact test was used to compare the enrichment of *MET* mutations between the age of diagnosis groups. Fisher's exact test p-values: * $p<0.05$, ** $p<0.01$, **** $p<0.0001$. NS (Never-smoker), LS (Light smoker), MS (Moderate smoker), HS (Heavy smoker), ES (Ever-smoker).

FIGURE S3

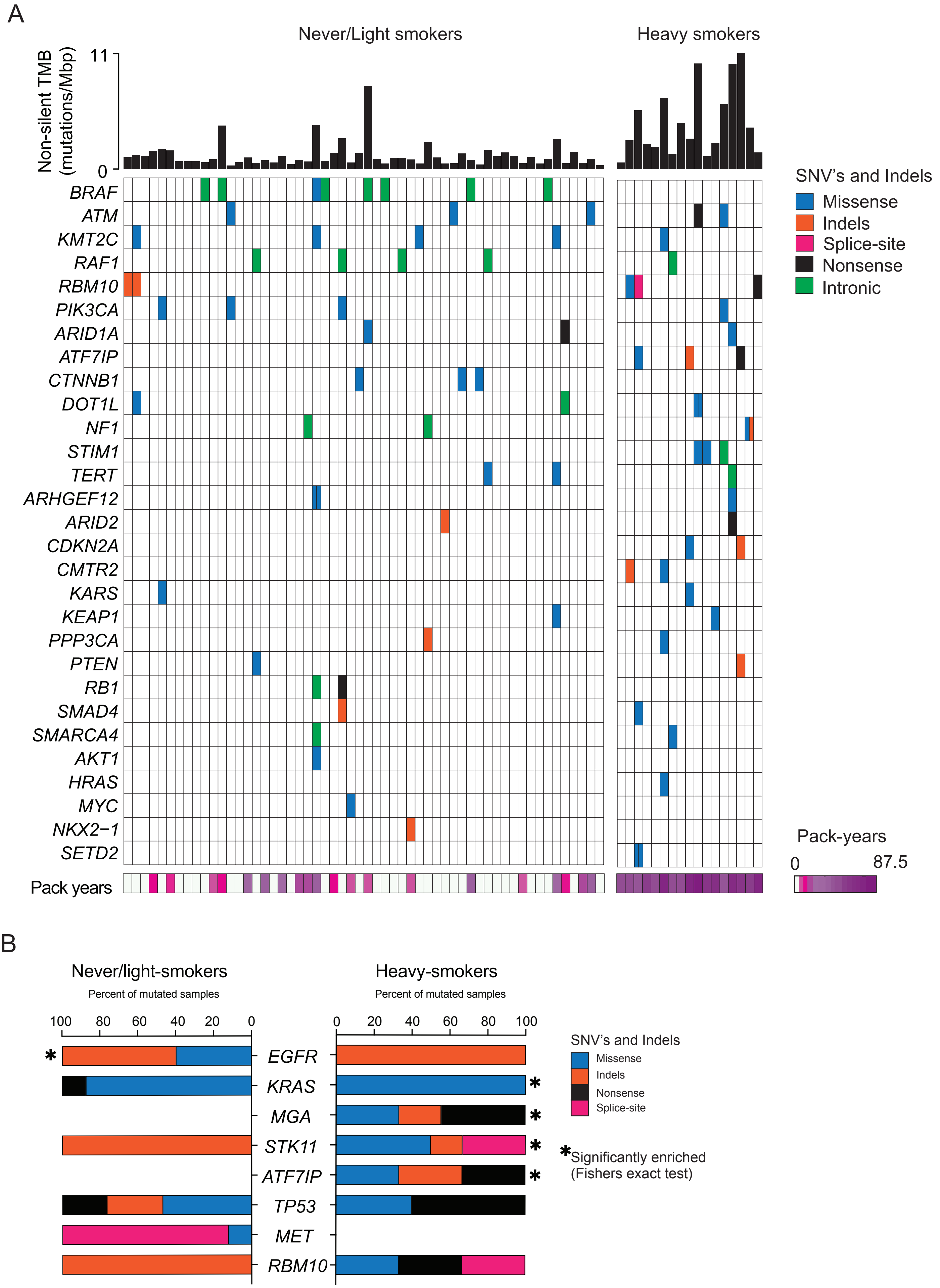
A
Never/Light smokers
Heavy smokers
Non-silent TMB (mutations/Mbp)
11
0
BRAF
ATM
KMT2C
RAF1
RBM10
PIK3CA
ARID1A
ATF7IP
CTNNB1
DOT1L
NF1
STIM1
TERT
ARHGEF12
ARID2
CDKN2A
CMTR2
KARS
KEAP1
PPP3CA
PTEN
RB1
SMAD4
SMARCA4
AKT1
HRAS
MYC
NKX2−1
SETD2
Pack years
SNV's and Indels
Missense
Indels
Splice-site
Nonsense
Intronic
Pack-years
0
87.5
B
Never/light-smokers
Percent of mutated samples
100
80
60
40
20
0
Heavy-smokers
Percent of mutated samples
0
20
40
60
80
100
EGFR
KRAS
MGA
STK11
ATF7IP
TP53
MET
RBM10
SNV's and Indels
Missense
Indels
Nonsense
Splice-site
*Significantly enriched
(Fishers exact test)

Figure S3. (**A)** Oncoplot of mutations belonging to the RTK/Ras/Raf pathway or genes known to be altered in lung adenocarcinoma from the WHI cohort. The top bar plot shows the non-silent TMB rate for each patient. **(B)** Percentage of types of mutations for key genes. Genes that were significantly enriched in one or the other smoking groups were marked by a star. Fisher's exact test was used to measure enrichment; the star represents enrichment in one smoking cohort over another ($p < 0.05$).

FIGURE S4

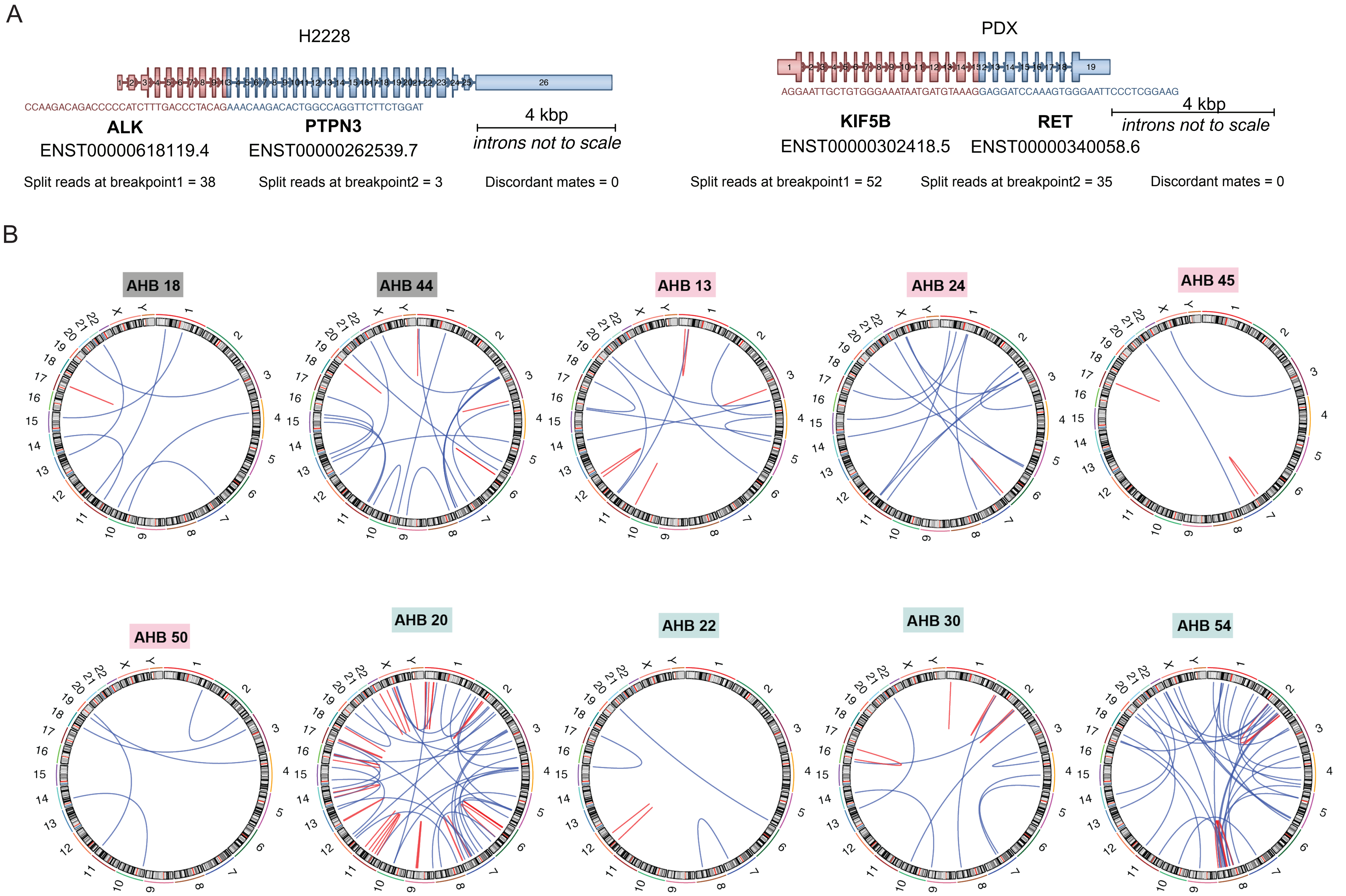
A
H2228
CCAAGACAGACCCCCATCTTTGACCCTACAGAAACAAGACACTGGCCAGGTTCTTCTGGAT
ALK
ENST00000618119.4
PTPN3
ENST00000262539.7
4 kbp
introns not to scale
Split reads at breakpoint1 = 38
Split reads at breakpoint2 = 3
Discordant mates = 0
PDX
AGGAATTGCTGTGGGAAATAATGATGTAAAGGAGGATCCAAAGTGGGAATTCCCTCGGAAG
KIF5B
ENST00000302418.5
RET
ENST00000340058.6
4 kbp
introns not to scale
Split reads at breakpoint1 = 52
Split reads at breakpoint2 = 35
Discordant mates = 0
B
AHB 18
AHB 44
AHB 13
AHB 24
AHB 45
AHB 50
AHB 20
AHB 22
AHB 30
AHB 54

Figure S4. (A) Schematic representation of translocations found in control cell line *H2228* and lung adenocarcinoma metastatic PDX (AHB001) sample. Schematic shows the location of regions involved in the translocation on the origin chromosomes, coverage of different regions of the genes involved, and the final structure of the translocation. **(B)** Circos plots showing inter-chromosomal fusions (blue) and intra-chromosomal fusion (red) for 10 oncogene-negative samples that underwent whole-genome sequencing. SvABA was used to determine translocations spanning lengths >10kb and where partners involved in the translocation were in protein-coding regions. Sample IDs are colored to represent the smoking history of the patients never-smokers (gray), light smokers (pink) and heavy smokers (green).

FIGURE S5

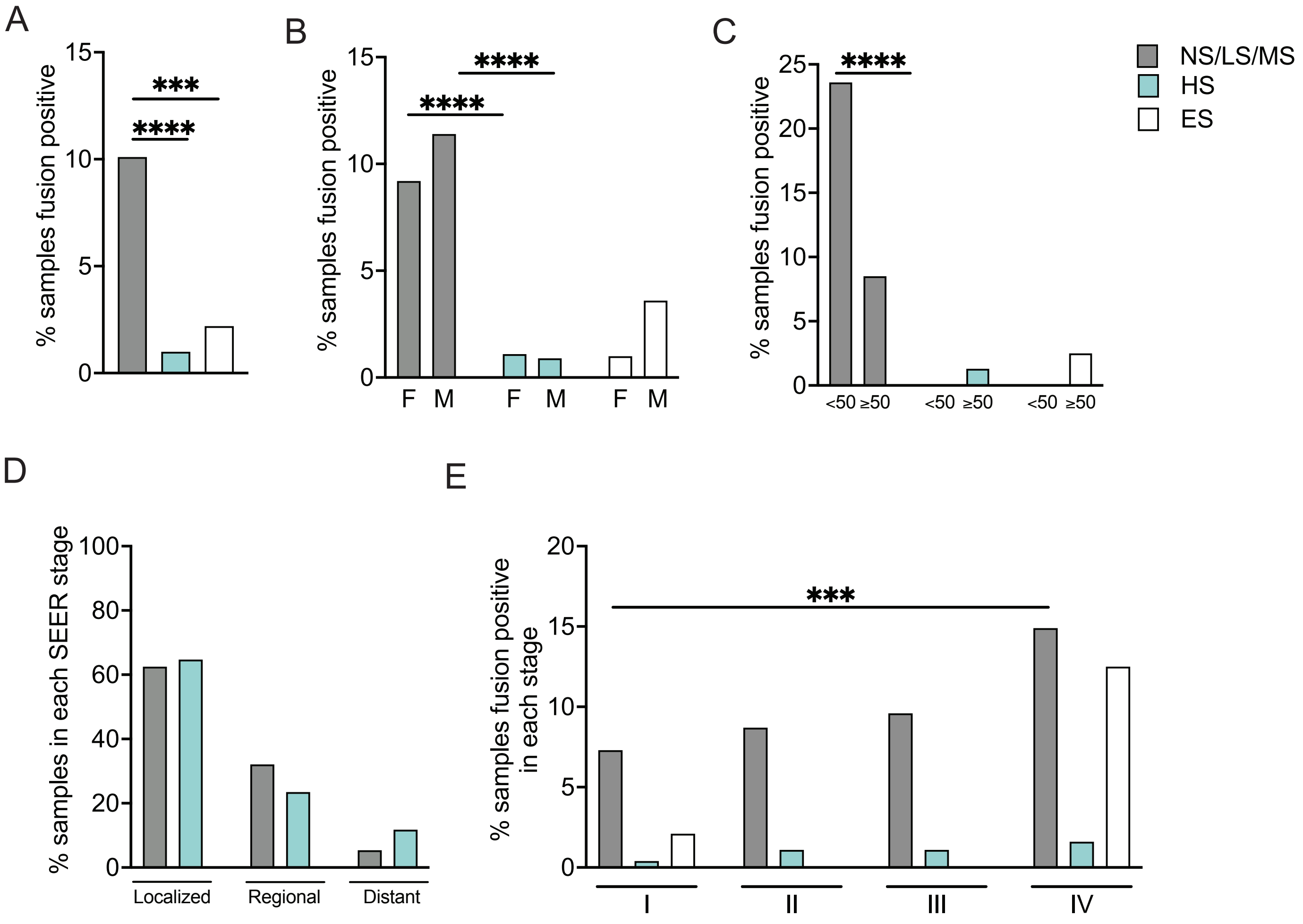

A
B
C
D
E
NS/LS/MS
HS
ES
% samples fusion positive
15
10
5
0
***
****
F
M
25
20
<50
≥50
% samples in each SEER stage
100
80
60
40
Localized
Regional
Distant
% samples fusion positive in each stage
I
II
III
IV

Figure S5. (A) Percent samples that are positive for *ALK*, *RET* and *ROS1* fusions from the analysis of external cohorts TCGA, MSK, OncoSG, Sherlock and Broad, by smoking status **(B)** Percent samples fusion-positive by sex and smoking status in the external cohorts. **(C)** Percent samples fusion-positive by age at diagnosis and smoking status in the external cohorts. **(D)** Percent samples in the WHI cohort that were diagnosed with tumors in the different SEER stage groups. **(E)** Percent samples fusion-positive by stage and smoking status in the external cohort. Fisher's exact test was used to calculate the enrichment between groups. Fisher's exact test p-values: *** $p<0.001$, **** $p<0.0001$. NS (Never-smoker), LS (Light smoker), MS (Moderate smoker), HS (Heavy smoker), ES (Ever-smoker).

FIGURE S6

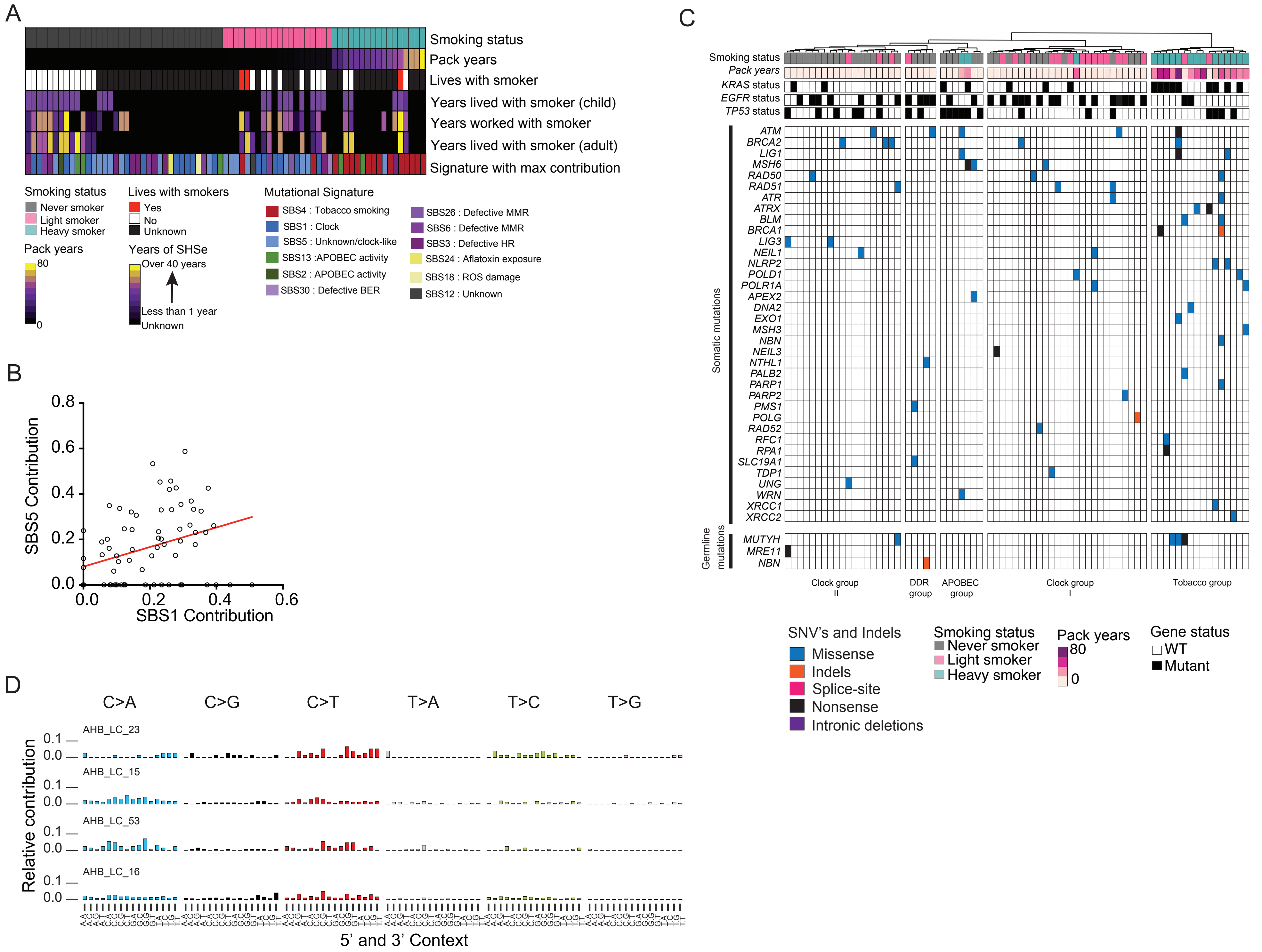
A
Smoking status
Pack years
Lives with smoker
Years lived with smoker (child)
Years worked with smoker
Years lived with smoker (adult)
Signature with max contribution
Smoking status
Never smoker
Light smoker
Heavy smoker
Pack years
80
0
Lives with smokers
Yes
No
Unknown
Years of SHSe
Over 40 years
Less than 1 year
Unknown
Mutational Signature
SBS4 : Tobacco smoking
SBS1 : Clock
SBS5 : Unknown/clock-like
SBS13 :APOBEC activity
SBS2 : APOBEC activity
SBS30 : Defective BER
SBS26 : Defective MMR
SBS6 : Defective MMR
SBS3 : Defective HR
SBS24 : Aflatoxin exposure
SBS18 : ROS damage
SBS12 : Unknown
B
SBS5 Contribution
SBS1 Contribution
0.0
0.2
0.4
0.6
0.8
C
Smoking status
Pack years
KRAS status
EGFR status
TP53 status
Somatic mutations
ATM
BRCA2
LIG1
MSH6
RAD50
RAD51
ATR
ATRX
BLM
BRCA1
LIG3
NEIL1
NLRP2
POLD1
POLR1A
APEX2
DNA2
EXO1
MSH3
NBN
NEIL3
NTHL1
PALB2
PARP1
PARP2
PMS1
POLG
RAD52
RFC1
RPA1
SLC19A1
TDP1
UNG
WRN
XRCC1
XRCC2
Germline mutations
MUTYH
MRE11
NBN
Clock group II
DDR group
APOBEC group
Clock group I
Tobacco group
SNV's and Indels
Missense
Indels
Splice-site
Nonsense
Intronic deletions
Smoking status
Never smoker
Light smoker
Heavy smoker
Pack years
80
0
Gene status
WT
Mutant
D
C>A
C>G
C>T
T>A
T>C
T>G
AHB_LC_23
AHB_LC_15
AHB_LC_53
AHB_LC_16
0.1
0.0
Relative contribution
5' and 3' Context

Figure S6. (A) Heatmap showing primary smoke exposure and second-hand smoke exposure (SHSe) for each patient. Samples are sorted based on increasing pack years. Also shown is the signature with the maximum contribution to the mutational signature burden. **(B)** Scatter plot for SBS1 versus SBS5 contributions per sample. Simple linear regression shows a significant association between SBS1 and SBS5 contributions in the WHI cohort; p = 0.0026. **(C)** Oncoplot for somatic and germline mutations in DNA damage and repair (DDR) genes. (**D)** Relative mutational profile in all possible 5' and 3' contexts in *MUTYH* mutated samples. Y-axis shows the relative contributions of each mutational context.

FIGURE S7

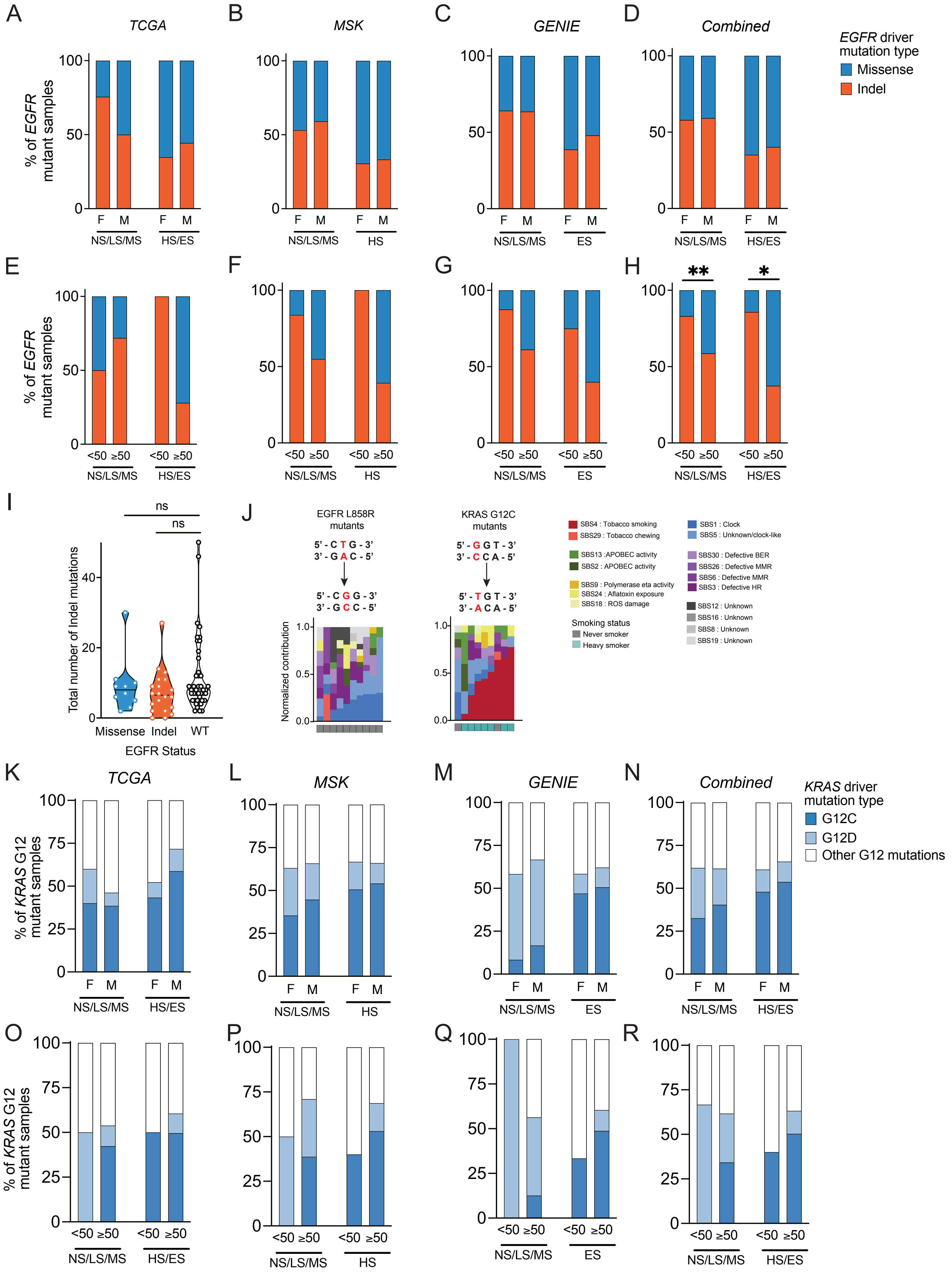
A
TCGA
% of EGFR mutant samples
F M F M
NS/LS/MS HS/ES
B
MSK
NS/LS/MS HS
C
GENIE
NS/LS/MS ES
D
Combined
NS/LS/MS HS/ES
EGFR driver mutation type
Missense
Indel
E
F
G
H
** *
<50 ≥50 <50 ≥50
I
ns
ns
Total number of Indel mutations
Missense Indel WT
EGFR Status
J
EGFR L858R mutants
5' - C T G - 3'
3' - G A C - 5'
5' - C G G - 3'
3' - G C C - 5'
KRAS G12C mutants
5' - G G T - 3'
3' - C C A - 5'
5' - T G T - 3'
3' - A C A - 5'
Normalized contribution
SBS4 : Tobacco smoking
SBS29 : Tobacco chewing
SBS13 :APOBEC activity
SBS2 : APOBEC activity
SBS9 : Polymerase eta activity
SBS24 : Aflatoxin exposure
SBS18 : ROS damage
Smoking status
Never smoker
Heavy smoker
SBS1 : Clock
SBS5 : Unknown/clock-like
SBS30 : Defective BER
SBS26 : Defective MMR
SBS6 : Defective MMR
SBS3 : Defective HR
SBS12 : Unknown
SBS16 : Unknown
SBS8 : Unknown
SBS19 : Unknown
K
TCGA
% of KRAS G12 mutant samples
L
MSK
M
GENIE
N
Combined
KRAS driver mutation type
G12C
G12D
Other G12 mutations
O
P
Q
R

Figure S7. (A-D) The percent of samples with either *EGFR* indel (orange) or missense (blue) mutations split by sex and smoking status for MSK (A), TCGA (B) and GENIE (C) cohorts and combined for all three cohorts (D). Two-tailed Fisher's test was used to measure enrichment of indel mutations **(E-H)** The percent of samples with either *EGFR* indel (orange) or missense (blue) mutations split by age at diagnosis and smoking status for MSK (E), TCGA (F) and GENIE (G) cohorts and combined for all three cohorts (H). Two-tailed Fisher's test was used to measure enrichment of indel mutations **(I)** Total number of indel mutations in the WHI cohort split by if they belonged to *EGFR* missense, indel or wild-type groups. Mann-Whitney test was used to compare the number of indel mutations to other groups. **(J)** Schematic of *EGFR* L858R mutation and *KRAS* G12C mutation at the nucleotide level (top). Mutational spectrum of *EGFR* and *KRAS* mutant samples in the WHI cohort with either L858R or G12C mutations only (bottom). **(K-N)** The percent of samples with either *KRAS* G12C mutations (dark blue) compared to G12D (light blue) and other G12 mutations (white) split by sex and smoking status for MSK (K), TCGA (L), GENIE (M) cohorts and all cohorts combine (N) Two-tailed Fisher's-exact test was used to compare number of KRAS G12C mutations in each group. **(O-R)** The percent of samples with either *KRAS* G12C mutations (dark blue) compared to G12D (light blue) and other G12 mutations(white) split by age at diagnosis and smoking status for MSK (O), TCGA (P), GENIE (Q) cohorts and all cohorts combine (R) Two-tailed Fisher's-exact test was used to compare number of *KRAS* G12C mutations in each group.

FIGURE S8

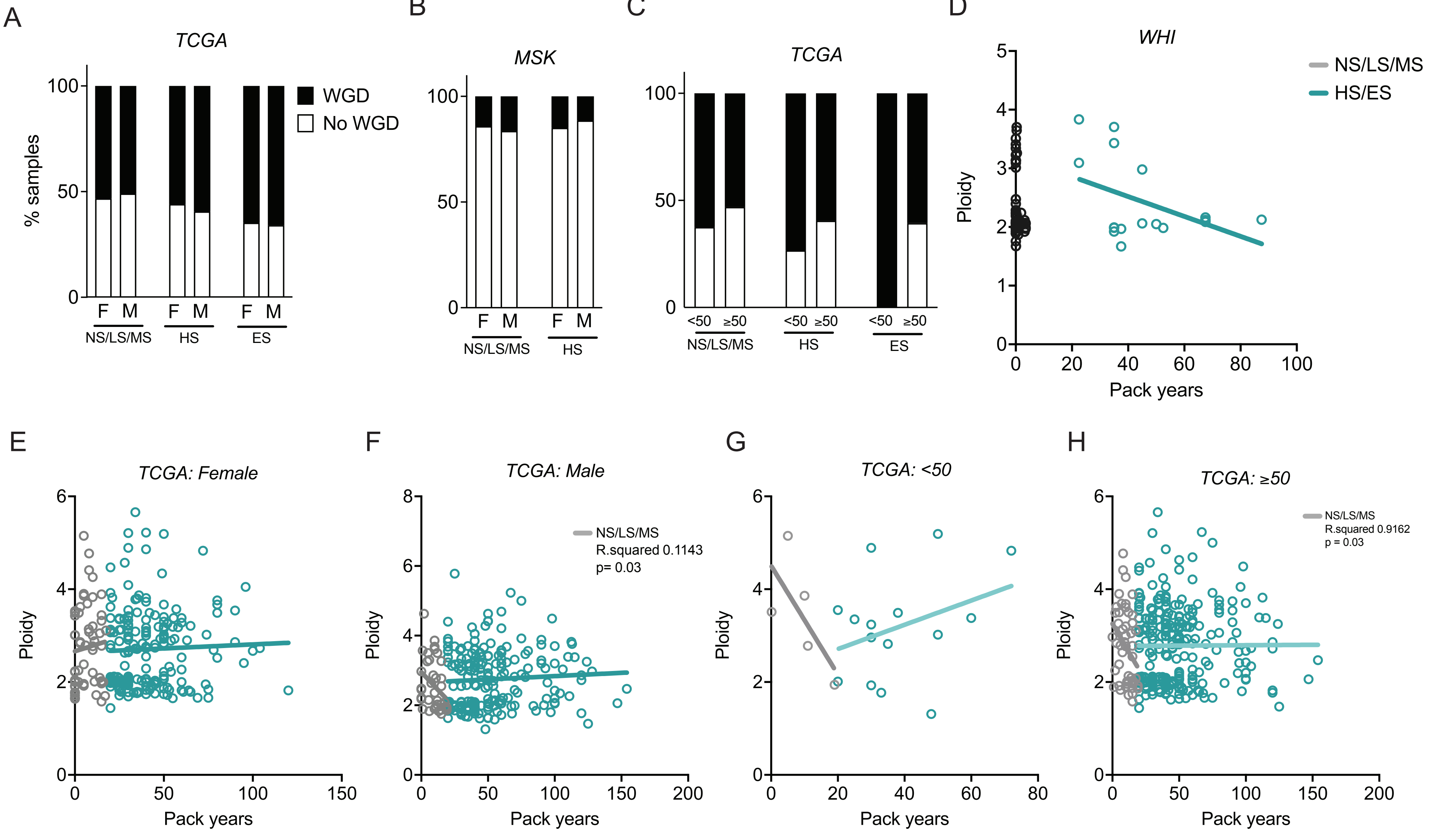

A
TCGA
% samples
100
50
0
WGD
No WGD
F M
NS/LS/MS
F M
HS
F M
ES
B
MSK
100
50
0
F M
NS/LS/MS
F M
HS
C
TCGA
100
50
0
<50 ≥50
NS/LS/MS
<50 ≥50
HS
<50 ≥50
ES
D
WHI
NS/LS/MS
HS/ES
Ploidy
Pack years
E
TCGA: Female
Ploidy
Pack years
F
TCGA: Male
NS/LS/MS
R.squared 0.1143
p= 0.03
Ploidy
Pack years
G
TCGA: <50
Ploidy
Pack years
H
TCGA: ≥50
NS/LS/MS
R.squared 0.9162
p = 0.03
Ploidy
Pack years

Figure S8. (A-B) Stacked bar graphs showing the percentage of samples with whole genome duplication (WGD) (black) or no WGD (white) in the TCGA (A) and MSK (B) cohorts. Enrichment of WGD was measured with a two-tailed Fisher's test. **(C)** Stacked bar graphs showing the percentage of samples with whole genome duplication (WGD) (black) or no WGD (white) in the TCGA cohort, split by age at diagnosis and grouped by smoking status. Enrichment of WGD was measured with a two-tailed Fisher's test **(D)** Scatterplot of ploidy versus pack-years cigarettes smoked in the WHI. **(E-H)** Scatterplot of ploidy versus pack-years cigarettes smoked in TCGA cohorts split by sex (E-F) or by age at diagnosis (G-H) Simple linear regression was used to identify any significant relationship between pack-years smoked and ploidy.

FIGURE S9

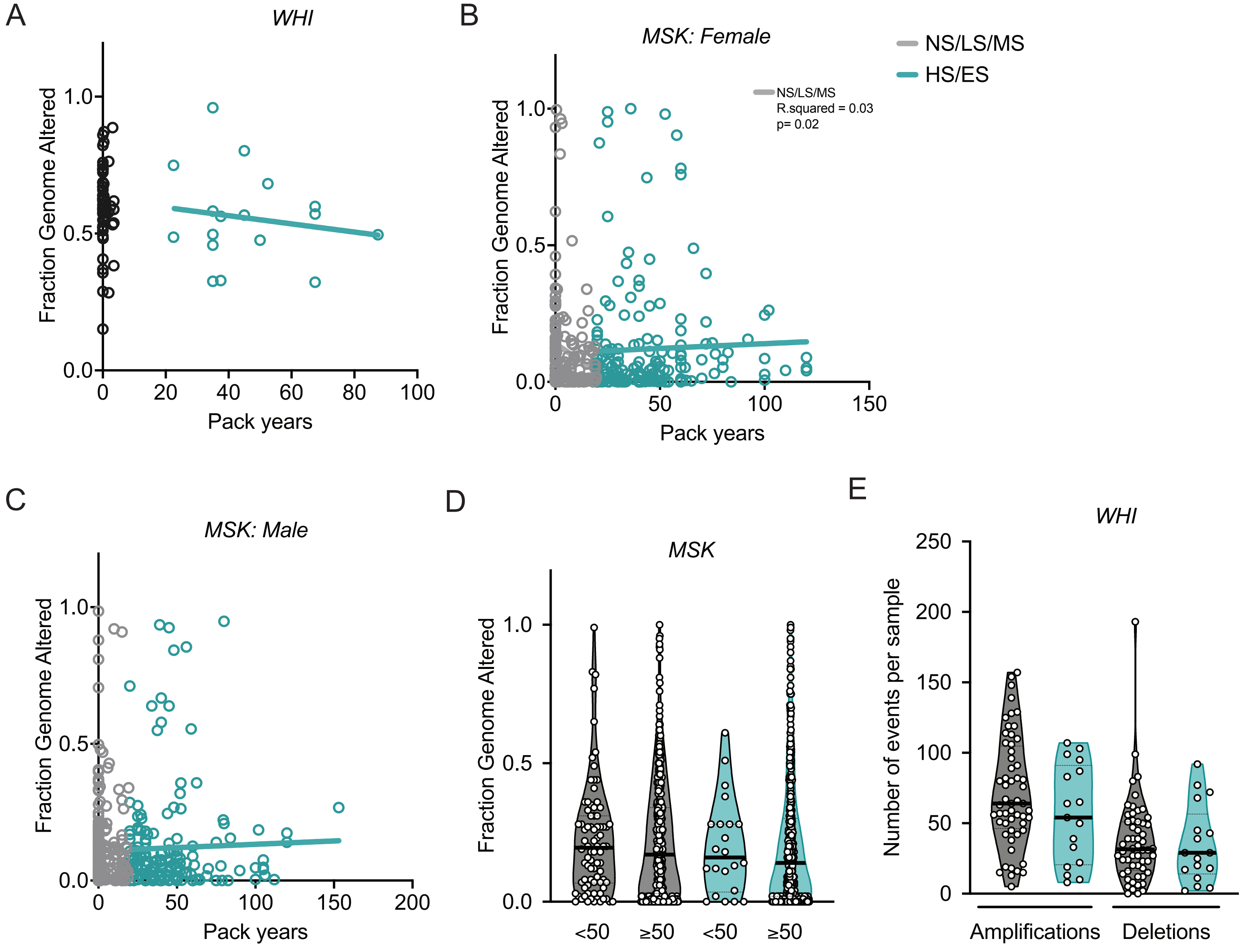

A
WHI
Fraction Genome Altered
Pack years
B
MSK: Female
NS/LS/MS
R.squared = 0.03
p= 0.02
Fraction Genome Altered
Pack years
NS/LS/MS
HS/ES
C
MSK: Male
Fraction Genome Altered
Pack years
D
MSK
Fraction Genome Altered
<50
≥50
<50
≥50
E
WHI
Number of events per sample
Amplifications
Deletions

Figure S9. (A-C) Scatterplot of fractions genome altered (FGA) versus pack-years cigarettes smoked in the WHI and MSK cohorts. For the MSK cohorts scatter plots are split by sex (B-C). Simple linear regression was used to identify any significant relationship between pack-years smoked and FGA. **(D)** Fraction genome altered in the MSK cohort split by smoking status and grouped by age at diagnosis. (**E)** The total number of amplifications and deletions in the WHI cohort in never-/light smokers and heavy smokers. Kruskal-Wallis/Dunns Test was used to test significance (D-E).
